# Lipidome modulation by dietary omega-3 polyunsaturated fatty acid supplementation or selective soluble epoxide hydrolase inhibition suppresses rough LPS-accelerated glomerulonephritis in lupus-prone mice

**DOI:** 10.3389/fimmu.2023.1124910

**Published:** 2023-02-16

**Authors:** Olivia K. Favor, Preeti S. Chauhan, Elham Pourmand, Angel M. Edwards, James G. Wagner, Ryan P. Lewandowski, Lauren K. Heine, Jack R. Harkema, Kin Sing Stephen Lee, James J. Pestka

**Affiliations:** ^1^ Department of Pharmacology and Toxicology, Michigan State University, East Lansing, MI, United States; ^2^ Institute for Integrative Toxicology, Michigan State University, East Lansing, MI, United States; ^3^ Department of Food Science and Human Nutrition, Michigan State University, East Lansing, MI, United States; ^4^ Department of Chemistry, Michigan State University, East Lansing, MI, United States; ^5^ Department of Pathobiology and Diagnostic Investigation, Michigan State University, East Lansing, MI, United States; ^6^ Department of Microbiology and Molecular Genetics, Michigan State University, East Lansing, MI, United States

**Keywords:** lupus, NZBWF1 mouse, soluble epoxide hydrolase, docosahexaenoic acid, glomerulonephritis, rough LPS, smooth LPS, epoxy fatty acid

## Abstract

**Introduction:**

Lipopolysaccharide (LPS)-accelerated autoimmune glomerulonephritis (GN) in NZBWF1 mice is a preclinical model potentially applicable for investigating lipidome-modulating interventions against lupus. LPS can be expressed as one of two chemotypes: smooth LPS (S-LPS) or rough LPS (R-LPS) which is devoid of O-antigen polysaccharide sidechain. Since these chemotypes differentially affect toll-like receptor 4 (TLR4)-mediated immune cell responses, these differences may influence GN induction.

**Methods:**

We initially compared the effects of subchronic intraperitoneal (i.p.) injection for 5 wk with 1) *Salmonella* S-LPS, 2) *Salmonella* R-LPS, or 3) saline vehicle (VEH) (Study 1) in female NZBWF1 mice. Based on the efficacy of R-LPS in inducing GN, we next used it to compare the impact of two lipidome-modulating interventions, ω-3 polyunsaturated fatty acid (PUFA) supplementation and soluble epoxide hydrolase (sEH) inhibition, on GN (Study 2). Specifically, effects of consuming ω-3 docosahexaenoic acid (DHA) (10 g/kg diet) and/or the sEH inhibitor 1-(4-trifluoro-methoxy-phenyl)-3-(1-propionylpiperidin-4-yl) urea (TPPU) (22.5 mg/kg diet ≈ 3 mg/kg/day) on R-LPS triggering were compared.

**Results:**

In Study 1, R-LPS induced robust elevations in blood urea nitrogen, proteinuria, and hematuria that were not evident in VEH- or S-LPS-treated mice. R-LPS-treated mice further exhibited kidney histopathology including robust hypertrophy, hyperplasia, thickened membranes, lymphocytic accumulation containing B and T cells, and glomerular IgG deposition consistent with GN that was not evident in VEH- or SLPS-treated groups. R-LPS but not S-LPS induced spleen enlargement with lymphoid hyperplasia and inflammatory cell recruitment in the liver. In Study 2, resultant blood fatty acid profiles and epoxy fatty acid concentrations reflected the anticipated DHA- and TPPU-mediated lipidome changes, respectively. The relative rank order of R-LPS-induced GN severity among groups fed experimental diets based on proteinuria, hematuria, histopathologic scoring, and glomerular IgG deposition was: VEH/CON< R-LPS/DHA ≈ R-LPS/TPPU<<< R-LPS/TPPU+DHA ≈ R-LPS/CON. In contrast, these interventions had modest-to- negligible effects on R-LPS-induced splenomegaly, plasma antibody responses, liver inflammation, and inflammation-associated kidney gene expression.

**Discussion:**

We show for the first time that absence of O-antigenic polysaccharide in R-LPS is critical to accelerated GN in lupus-prone mice. Furthermore, intervention by lipidome modulation through DHA feeding or sEH inhibition suppressed R-LPS-induced GN; however, these ameliorative effects were greatly diminished upon combining the treatments.

## Introduction

Systemic lupus erythematosus (lupus) is a complex, debilitating autoimmune disease that affects primarily women of childbearing age, attacks multiple organ systems, and features repeated cycles of remission and relapse (1). Lupus development and progression are associated with chronic inflammation, aberrant accumulation of dead/dying cells, release of autoantigens (AAgs) that promote T and B cell hyperactivation, and aberrant autoantibody (AAb) production (2, 3). Resultant AAb:AAg immune complex formation and peripheral tissue deposition activate the complement system and trigger infiltration of innate immune cells that subsequently secrete cytokines and chemokines. Collectively, these events promote a perpetual cycle of immune cell infiltration, proinflammatory mediator release, and cell death evoking unresolvable inflammation, further activation of autoreactive lymphocytes, and irreversible tissue damage (4, 5). Immune complex deposition in the kidneys of patients with lupus can lead to glomerulonephritis (GN) that progresses over time to end-stage kidney disease.

While genetic predilection is a primary contributor to lupus, its onset and progression can be potentiated or attenuated by environmental influences (6, 7). There is increasing recognition that exposure of individuals with lupus to infectious bacteria can trigger inflammation and activation of autoreactive lymphocytes *via* pathogen-associated molecular patterns (PAMPs), leading to exacerbation of lupus symptoms (8). In particular, exposure to Gram-negative bacteria through infection or gut leakage is common and could contribute to lupus flaring (6, 9–11). Lipopolysaccharide (LPS) is an important structural component of the Gram-negative bacterial cell wall that binds toll-like receptor 4 (TLR4) on innate and adaptive immune cells to promote nuclear translocation of NF-κB, which upregulates expression of genes that contribute to autoimmune disease progression (12–15). Consistent with this premise, earlier preclinical investigations have reported that repeated LPS exposure elicits autoimmune disease in non-autoimmune BALB/c and C57BL/6 (C57) mice (16–19), and, furthermore, accelerates spontaneous autoimmunity in lupus-prone New Zealand Black/White F1 (NZBWF1), MRL/lpr (MRL) and BXSB mice (20–25). Key mechanisms that have been proposed for LPS-accelerated autoimmune disease include induction of polyclonal B-cell activation, decreased immune complex uptake by mononuclear phagocytes, delayed clearance of circulating immune complexes, and increased immune complex deposition in the kidney (16–25).

Since there is no cure for lupus, it is managed in the clinic through a variety of prescribed pharmaceuticals, such as glucocorticoids, immunosuppressants, and monoclonal antibodies (26, 27). Despite the efficacy of these therapeutics against chronic inflammation and autoreactive immunity, patients still incur drug-related adverse side effects and steep financial costs (28). In addition, these therapeutics might need to be taken indefinitely because lupus symptoms can flare spontaneously over a lifetime. Therefore, there is a critical need for safer, more cost-effective interventions against lupus onset and progression.

One intervention of potential high relevance to lupus is modulation of the lipidome by dietary supplementation with marine ω-3 polyunsaturated fatty acids (PUFAs). Numerous clinical and preclinical studies have demonstrated that increasing consumption the ω-3 PUFAs docosahexaenoic acid (DHA) and eicosapentaenoic acid (EPA) at the expense of terrestrial ω-6 PUFAs like linoleic acid (LA) and arachidonic acid (ARA) has potential benefits for reducing severity of chronic inflammatory diseases (reviewed in (29)), including autoimmune diseases like lupus (30–33). Beneficial effects of ω-3 PUFAs are linked to: 1) reduced production of proinflammatory ω-6 metabolites, 2) generation of specialized pro-resolving mediators, 3) changes in membrane structure/function, and 4) modulation of gene expression by altering G-protein-couple receptor signaling and transcription factor activity (29).

Another possible lipidome-mediated intervention for lupus is to modulate the lipidome by pharmacological inhibition of soluble epoxide hydrolase (sEH). Among the sEH inhibitors employed in preclinical studies, 1-(4-trifluoro-methoxy-phenyl)-3-(1-propionylpiperidin-4-yl) urea (TPPU) is highly preferred because it is safe and exhibits impressive potency, biological activity, and pharmacokinetic distribution (34–36) without evident non-specific binding (37). TPPU has been shown to be efficacious in preclinical studies at reducing pathogenesis of many chronic inflammatory diseases (38) and more recently, autoimmune diseases including lupus GN (39), autoimmune encephalitis (40), and rheumatoid arthritis (41). One mode of action for TPPU and other sEH inhibitors is believed to involve skewing of the cytochrome P450 (CYP) ω-6 metabolite profile to favor anti-inflammatory/pro-resolving epoxy fatty acids (EpFAs) over proinflammatory or less active dihydroxy fatty acids (DiHFAs; vicinal diols) generated because of sEH activity. Furthermore, there is intriguing but limited evidence in preclinical models that suggests there is enhanced efficacy in anti-inflammatory effects when ω-3s are combined with pharmacologic inhibition of sEH (42–46).

Preclinical animal models are integral for investigating new therapies for managing lupus progression and resultant GN (47). Over the past decade, LPS-accelerated severe lupus GN in NZBWF1 mice has been extensively used to explore efficacy of a wide spectrum of novel interventions including Tris dipalladium (48), epigallocatechin-3-gallate (49), traditional Chinese medicinal herbs (50), citral (51), gisenoside (52), honokial (53), antroquinonol (49), and xenon (54), suggesting that this model might be similarly amenable for addressing effects of lipidome modulation through dietary supplementation or pharmacotherapy. However, one caveat to the use of the LPS-accelerated GN as a preclinical model is the lack of clarity on how different LPS chemotypes influence the GN response. LPS is comprised of three moieties linked by covalent bonds: i) lipid A, ii) rough core oligosaccharide, and iii) O-antigenic polysaccharide side chain which determines serotype (55). Importantly, environmental stimuli and genetic mutations can cause Gram-negative bacteria to synthesize LPS with variable polysaccharide lengths *via* outer membrane remodeling (56). While smooth LPS (S-LPS) includes the O-antigenic side chain, rough LPS (R-LPS) lacks the side chain completely or, in some cases, contains portions of the rough core oligosaccharide. Clinically relevant Gram-negative bacteria typically express S-LPS; however, some heterogeneously co-express R-LPS of varying lengths (57, 58). Significantly, the mechanisms by which these two chemotypes activate TLR4 are very different. It has been demonstrated that R-LPS can efficiently activate TLR4 on both CD14^+^ and CD14^-^ cells as compared to S-LPS which acts primarily on CD14^+^ cells (57, 59). These differences in TLR4 activation between the two chemotypes may influence their capacity to accelerate GN in NZBWF1 mice. However, while some investigations of LPS-accelerated murine GN explicitly specify using R-LPS, typically from *Salmonella* (16–18, 20–25), many others do not report the LPS chemotype used (23, 48–54, 60). Importantly, there has never been a head-to-head comparison of S-LPS and R-LPS accelerating GN in lupus-prone mice.

To address the research questions described above, we conducted two studies in lupus-prone female NZBWF1 mice. In Study 1, we compared the effects of R-LPS and S-LPS on GN induction to clarify how the presence or absence of O antigen polysaccharide impacts this widely used preclinical model. The results indicated that repeated injection with R-LPS accelerated severe GN whereas repeated injection with S-LPS did not. In Study 2, we evaluated how dietary DHA supplementation and/or pharmacologic inhibition of sEH influence R-LPS-accelerated GN. We found that DHA consumption and sEH inhibition alone suppressed GN, but the ameliorative effects of these interventions were lessened upon combining the treatments.

## Materials and methods

### Animals

The Institutional Animal Care and Use Committee at Michigan State University (MSU) approved all experimental protocols (AUF #201800113) in accordance with guidelines established by the National Institutes of Health. Six-week-old female lupus-prone NZBWF1 mice were procured from the Jackson Laboratory (Bar Harbor, ME) and randomized into experimental groups for each study (**Supplementary Tables 1**, **2**). Only female NZBWF1 mice were used in this study because female mice of this strain exhibit greater severity and prevalence of lupus-related symptoms (e.g., elevated antinuclear antibody titers, formation of immune complexes, glomerulonephritis) compared to male NZBWF1 mice (61). Mice were housed 2 or 4 per cage in Study 1 and 4 per cage in Study 2, and all mice were given free access to food and water. Consistent lighting (12 h light/dark cycle), temperature (21-24°C), and humidity (40-55%) were maintained in animal housing facilities.

### Diets

Four defined diet formulations were prepared as described in **Supplementary Table 3**. All formulations used purified American Institute of Nutrition (AIN)-93G diet (70 g/kg fat) as a base to provide optimal nutrition to experimental rodents (62). All diets contained 10 g/kg corn oil as a source of essential ω-6 fatty acids. The basal diet for Study 1 and control (CON) diet for Study 2 contained 60 g/kg high-oleic safflower oil (Hain Pure Food, Boulder, CO). For DHA-enriched diets, human equivalent caloric consumption of 5 g DHA per day was achieved by adding 25 g/kg microalgal oil containing 40% DHA (DHASCO; DSM Nutritional Products, Columbia, MD) in place of high-oleic safflower oil, resulting in 10 g DHA/kg diet (63). For TPPU-amended diets, 22.5 mg TPPU (95% purity based on H-NMR analysis), synthesized and purified as described previously (34), was added to 1 kg of CON or DHA diet, resulting in the TPPU and TPPU+DHA diets. Fatty acid (**Table 1**) and TPPU (**Supplementary Table 4**) concentrations in each diet were confirmed as described below.

**Table 1:** Experimental diet fatty acid concentrations.

Common name	Chemical formula	CON	DHA	TPPU	TPPU+DHA
Capric	C10:0	<LOD	0.30 ± 0.07	<LOD	0.25 ± 0.06
Lauric	C12:0	0.02 ± 0.00	1.67 ± 0.11	0.01 ± 0.00	1.60 ± 0.06
Myristic	C14:0	0.09 ± 0.00	4.97 ± 0.19	0.10 ± 0.00	4.79 ± 0.02
Myristoleic	C14:1	<LOD	0.08 ± 0.00	<LOD	0.09 ± 0.01
Pentadecanoic	C15:0	0.02 ± 0.00	0.01 ± 0.00	0.01 ± 0.00	0.01 ± 0.00
Palmitic	C16:0	6.29 ± 0.19	8.63 ± 0.22	6.50 ± 0.02	8.22 ± 0.05
Palmitoleic	C16:1ω7	0.02 ± 0.01	0.02 ± 0.01	0.02 ± 0.00	0.01 ± 0.00
Hygogeic	C16:1ω9	0.08 ± 0.01	0.91 ± 0.08	0.08 ± 0.01	0.99 ± 0.03
Heptadecanoic	C17:0	0.02 ± 0.00	0.02 ± 0.00	0.03 ± 0.00	0.02 ± 0.00
Stearic	C18:0	1.92 ± 0.07	1.55 ± 0.02	1.96 ± 0.07	1.57 ± 0.03
Vaccenic	C18:1ω7	0.81 ± 0.01	0.54 ± 0.01	0.76 ± 0.02	0.53 ± 0.01
Oleic	C18:1ω9	70.38 ± 0.63	50.85 ± 0.86	69.60 ± 0.90	51.98 ± 0.16
Linoleic	C18:2ω6	19.05 ± 0.43	16.16 ± 0.78	19.62 ± 0.93	15.14 ± 0.24
Alpha-linolenic	C18:3ω3	0.24 ± 0.02	0.20 ± 0.02	0.25 ± 0.02	0.19 ± 0.01
Arachidic	C20:0	0.36 ± 0.02	0.27 ± 0.01	0.35 ± 0.02	0.26 ± 0.01
Eicosenoic	C20:1ω9	0.27 ± 0.01	0.17 ± 0.02	0.27 ± 0.02	0.17 ± 0.02
Arachidonic	C20:4ω6	<LOD	<LOD	<LOD	<LOD
Behenic	C22:0	0.24 ± 0.02	0.19 ± 0.02	0.25 ± 0.03	0.20 ± 0.02
Eicosapentaenoic	C20:5ω3	<LOD	<LOD	<LOD	<LOD
Docosapentaenoic ω-3	C22:5ω3	<LOD	0.22 ± 0.03	<LOD	0.24 ± 0.01
Docosahexaenoic	C22:6ω3	<LOD	13.12 ± 0.39	<LOD	13.61 ± 0.19
Lignoceric	C24:0	0.13 ± 0.01	0.10 ± 0.01	0.14 ± 0.02	0.11 ± 0.01
Nervonic	C24:1ω9	0.08 ± 0.01	0.02 ± 0.01	0.05 ± 0.02	0.03 ± 0.01
Σ SFA		9.08 ± 0.29	17.71 ± 0.52	9.34 ± 0.11	17.02 ± 0.10
Σ MUFA		71.64 ± 0.65	52.59 ± 0.92	70.79 ± 0.88	53.80 ± 0.14
Σ PUFA		19.29 ± 0.42	29.70 ± 0.68	19.87 ± 0.95	29.18 ± 0.08
Σ ω-6 PUFA		19.05 ± 0.43	16.16 ± 0.78	19.62 ± 0.93	15.14 ± 0.24
Σ ω-3 PUFA		0.24 ± 0.02	13.54 ± 0.44	0.25 ± 0.02	14.04 ± 0.19
ω6/ω3 ratio		82.28 ± 9.13	1.20 ± 0.08	78.73 ± 3.51	1.08 ± 0.03

Data are presented as percent of total fatty acids (mean ± SEM, n = 8/gp) as measured by GC-MS. LOD, limit of detection.

### Dietary fatty acid analyses

Fatty acid composition in each experimental diet was determined by modifying a previously described protocol (64). Briefly, 400 mg of each diet sample was reconstituted in a 4:1 (v/v) ethanol/methanol solution + 0.1% (v/v) butylated hydroxytoluene (BHT) and heated 15 min at 55°C in a CEM Mars 6 Xpress microwave digestion system (CEM Corporation, Matthews, NC). Then, 2 mg of extracted fatty acids from each diet sample were converted to fatty acid methyl esters (FAMEs) by treating with 500 µl of toluene and 20 µg of internal standard (methyl-12-tridecenoate), incubating with 2 ml of KOH (0.5 N) at 50°C for 10 min, then subsequently incubating with 3 ml of methanolic HCl (5% [v/v] at 80°C for 10 min to allow base-catalyzed methylation and acid-catalyzed methylation, respectively. Following methylation, 2 ml of HPLC-grade water was added to the samples, and FAMEs were extracted by adding 2 ml of hexane to the samples twice. Extracted FAMEs were dried under nitrogen with an Organomation Multivap Nitrogen Evaporator (Organomation Associates, Berlin, MA). Dried FAMEs were then resuspended in 1 ml of isooctane and kept at -20°C until further analysis.

FAMEs were analyzed by GC-MS as previously described (64). Briefly, FAMEs in each sample were separated on a Perkin Elmer 680/600 GC-MS (Waltham, MA) outfitted with a HP-88 capillary column (100 m × 0.25 mm inner diameter × 0.2 µm film thickness; Agilent Technologies, Santa Clara, CA). MassLynx^TM^ (4.1 SCN 714; Waters Corporation, Milford, MA) was used to compare analyte retention time and electron ionization (EI) mass fragmentation to those in the reference standard, which consisted of Supelco 37 Component FAME Mix (Sigma-Aldrich, St. Louis, MO), mead acid, docosatetraenoic acid, ω-3 docosapentaenoic acid (DPA), ω-6 DPA, and palmitelaidic acid (Cayman Chemical, Ann Arbor, MI). FAME analyte peak areas were converted to individual FAME concentrations using a standard curve based on the reference standard and internal standard. For fatty acids with a detected chain length of 10 to 24 carbon atoms, fatty acid content in the diet is reported as percentage (w/w) of total fatty acids quantified (**Table 1**).

### LPS preparation

S-LPS from *Salmonella enterica* serotype minnesota (cat. #L6261) and R-LPS from *Salmonella enterica* serotype minnesota Re 595 (cat. #L9724) were purchased from Sigma Aldrich (St. Louis, MO). Immediately prior to all intraperitoneal (i.p.) injections, stock suspensions of LPS were prepared in sterile phosphate buffered saline (PBS), sonicated for 15 min, and vortexed for 1 min.

### Experimental design

**Figure 1:**
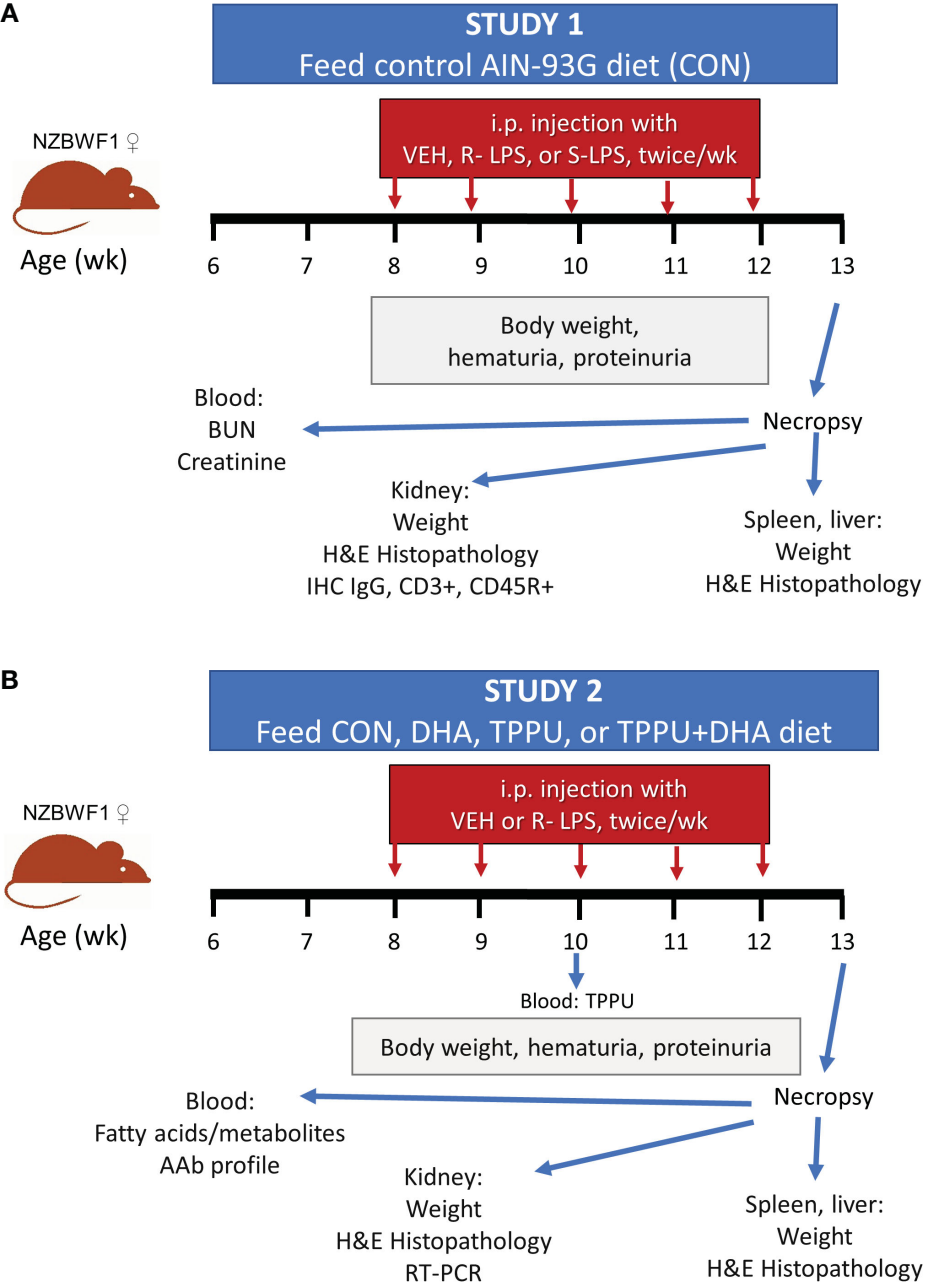
Experimental design for Study 1 **(A)** and Study 2 **(B)**. **(A)** At 6 wk of age, female NZBWF1 mice (*n =* 2-4/gp) were placed on CON diet. Beginning at 8 wk of age, mice were injected interperitoneally twice per wk for 5 wk with 500 µl of PBS vehicle, 0.8 µg/g body weight (BW) S-LPS, or 0.8 µg/g BW R-LPS. Mice were sacrificed at 13 wk of age, or 5 wk after the first LPS injection. **(B)** At 6 wk of age, female NZBWF1 mice (*n =* 8/gp) were placed on CON diet, DHA diet, TPPU diet, or TPPU+DHA diet. Beginning at 8 wk of age, mice were injected interperitoneally twice per wk for 5 wk with 500 µl of PBS vehicle or 0.6 µg/g BW R-LPS. Mice were sacrificed at 13 wk of age, or 5 wk after the first LPS injection.

Experimental designs for Study 1 and Study 2 are shown in **Figures 1A, B**, respectively. In both studies, female lupus-prone mice were administered experimental diets beginning at age 6 wk and maintained on the same diets throughout the entire experiment. To prevent lipid oxidation, all experimental diets were prepared every other week and stored at -20°C until administered to mice. Mice received fresh diet every day. Starting at age 8 wk, all groups of mice were injected intraperitoneally with S-LPS, S-LPS, or PBS vehicle twice per wk for 5 wk, for 10 total injections. On a weekly basis, body weights were measured and urine sampled for development of proteinuria and hematuria using clinical protein dipsticks (Cortez Diagnostics, Calabasas, CA) and blood dipsticks (Teco Diagnostics, Anaheim, CA), respectively. To compare the inflammatory and autoimmune responses triggered by S-LPS and R-LPS (Study 1), groups of female mice (n = 2-4/gp) were given control (CON) AIN-93G diet and intraperitoneally injected with S-LPS (0.8 µg/g body weight [BW]) or R-LPS (0.8 µg/g BW) in 500 µl of PBS or PBS vehicle as previously described (52). To assess effects of separate and concurrent DHA and TPPU administration on lupus GN induced by R-LPS (Study 2), female mice (n = 8/gp) were fed one of four experimental diets: 1) CON, 2) DHA, 3) TPPU, or 4) TPPU+DHA. Mice were intraperitoneally injected with R-LPS (0.6 µg/g BW) in 500 µl of PBS or PBS vehicle as previously described (53). After 5 R-LPS injections, blood samples were collected from the lateral saphenous vein to assess TPPU plasma concentration (Study 2). Mice for both Study 1 and Study 2 were terminated at age 13 wk (5 wk after the first LPS injection). This timepoint was selected for termination because it corresponds with development of accelerated, severe lupus GN previously reported in female NZBWF1 mice (48, 52, 53).

### Necropsy and tissue collection

Primary euthanasia for all mice occurred by intraperitoneal injection of 56 mg/kg BW sodium pentobarbital, followed by abdominal aortic exsanguination as a means of secondary euthanasia. Blood was obtained with heparin-coated syringes and plasma isolated by centrifugation at 3500 *x* g for 10 min under cold conditions (4°C). An antioxidant cocktail (0.2 mg/ml butylated hydroxytoluene, 0.2 mg/ml triphenylphosphine, 0.6 mg/ml EDTA) (65) was prepared and added at a 5% (v/v) concentration to all plasma aliquots designated for LC-MS/MS analysis. All plasma samples were stored at -80°C as single-use aliquots for LC-MS/MS, blood urea nitrogen (BUN) and creatinine quantification, and AAb microarray profiling. The left kidney was removed and fixed in 10% (v/v) neutral-buffered formalin (Fisher Scientific, Pittsburgh, PA) for 24 h. The right kidney was cut longitudinally, with one half immersed in RNAlater (Thermo Fisher Scientific, Waltham, MA) overnight at 4°C then stored at –80°C for RNA analysis. The spleen was transversely cut in half, with one half fixed in 10% formalin and the other half immersed in RNAlater as described above. The left lateral lobe of the liver was cut transversely, with one half of the lobe fixed in 10% formalin fixative and the other half immersed in RNAlater as described above. All fixed tissues were transferred to 30% (v/v) ethanol for additional routine processing, for light microscopic examination, and for long-term storage.

### Red blood cell fatty acid analysis

Red blood cell samples were sent to OmegaQuant Inc. (Sioux Falls, SD) for determination of membrane fatty acid concentrations by gas-liquid chromatography (GLC) as previously described (63).

### LC-MS/MS quantitation of plasma TPPU and oxylipins

Waters Oasis-HLB cartridges (part WAT094226, lot 176A30323A) were used for sample preparation and clean-up purposes. Solid-phase extraction (SPE) cartridges were prepared for solid phase extraction by washing once with 2 ml of ethyl acetate, twice with 2 ml of methanol, and twice with 2 ml of 95:5 (v/v) water/methanol + 0.1% (v/v) acetic acid. Plasma was then loaded onto the cartridges, and samples were spiked with 10 μl of deuterated internal standard solution (16 nM BGB2-d4, 10 nM LTB4-d4, 16 nM 8,9-DiHETrE-d11, 16 nM 9-HODE-d4, 20 nM 15(S)-HETE-d8, 40 nM 5(S)-HETE-d8, 40 nM 8,9-EpETrE-d11) and 10 μl of antioxidant cocktail (0.2 mg/ml butylated hydroxytoluene, 0.2 mg/ml triphenylphosphine, 0.6 mg/ml EDTA). After loading samples, cartridges were washed with 1.5 ml of 95:5 (v/v) water/methanol + 0.1% (v/v) acetic acid then dried with a low vacuum for 20 min to remove water and other unwanted residues. For elution, 6 µl of trap solution (30% [v/v] glycerol in methanol) was added to separate 2-ml Eppendorf tubes, then SPE cartridges were washed with 0.5 ml of methanol followed by 1 ml of ethyl acetate. The eluents were then concentrated under a high vacuum, and residues were reconstituted in 100 μl of 75% ethanol (v/v) containing 10 nM 12-[[(cyclohexylamino)carbonyl]amino]-dodecanoic acid (CUDA) as an internal standard. The samples then vortexed for 5 min followed by filtration through a 0.45-μm filter, then the filtrates were transferred to LC-MS/MS vials for analysis.

A XBridge BEH C18 2.1x150 mm, 5 µm, HPLC column, (ser. #01723829118314) was used for ultra-performance liquid chromatography (UPLC). The column was connected to a Waters TQ-XS tandem quadrupole UPLC/MS/MS instrument outfitted with a Waters ACQUITY SDS pump and Waters ACQUITY CM detector (Milford, MA). For UPLC, the chromatographic method was optimized to separate all analytes in 20 min using a sample volume of 10 µl and flow rate of 250 µl/min (**Supplementary Table 5**). Gradient elution was performed by using 0.1% (v/v) acetic acid in water for mobile phase A and 84:16 (v/v) acetonitrile/methanol + 0.1% glacial acetic acid for mobile phase B. During sample injection, the Waters ACQUITY FTN autosampler (Milford, MA) was held at a consistent temperature of 10°C.

The ionization source for multiple reaction monitoring (MRM) modes was electrospray. MRM transitions and source parameters were optimized for each standard compound by individually infusing each compound separately into the mass spectrometer, ultimately to achieve the most optimal selectivity and sensitivity. For each experimental sample, Waters MassLynx™ MS software v4 (Milford, MA) was used to quantify analyte area, internal standard (IS) area, raw concentration (in nM), and signal-to-noise (S/N) ratio based on an 8spots-calibration linear standard curve. Dilution factors were calculated for each sample by dividing the original sample volume (in µl) by 100 µl. Normalized analyte concentrations in each sample were then quantified by dividing raw analyte concentrations by the sample’s corresponding dilution factor.

### Plasma BUN and creatinine quantification

Plasma levels of BUN and creatinine were quantified using a Urea Nitrogen Colorimetric Detection Kit (Thermo Fisher Scientific, Waltham, MA; cat. #EIABUN) and Creatinine Colorimetric Assay Kit (Cayman Chemical, Ann Arbor, MI; cat. #700460), respectively, according to the manufacturers’ instructions.

### Histopathology of kidney, spleen, and liver

Formalin-fixed kidneys were embedded in paraffin, sectioned at a thickness of 5 µm, and stained with hematoxylin and eosin (H&E) or Periodic acid Schiff and hematoxylin (PASH). A board-certified veterinary pathologist semi-quantitatively scored sectioned tissues in a blinded manner (i.e., without knowledge of individual animal treatments) using a modification of the International Society of Nephrology/Renal Pathology Society (ISN/RPS) classification system for lupus GN (66). Each tissue section was assigned one of the following grades (0): normal glomeruli and no tubular proteinosis (1); multifocal segmental proliferative GN with mild tubular proteinosis and occasional early glomerular sclerosis and crescent formation (2); diffuse segmental proliferative GN with moderate tubular proteinosis, early glomerular sclerosis, and crescent formation; or (3) pervasive global proliferative and sclerosing GN with marked tubular proteinosis.

Fixed spleens and livers were processed and semi-quantitatively scored for histopathological development in a similar manner as the kidneys in this study. Scored liver lesions included (1) hepatocellular small and large vacuoles resembling lipid droplets and (2) periportal cellular inflammation (consisting primarily of inflammatory lymphocytes, plasma cells, and occasional neutrophils). Severity scores for these hepatic lesions were based on the percentage of the liver tissue section affected: (0) no treatment-induced lesions, (1) minimal (<10% affected), (2) mild (11-25% affected), (3) moderate (26-50% affected), (4) marked (51-75% affected), or (5) severe (76-100% affected).

### Kidney immunohistochemistry for IgG deposition and accumulation of T and B lymphocytes

Kidney immunohistochemistry was performed as previously described (67). Briefly, formalin-fixed kidney sections were stained with polyclonal goat anti-mouse IgG antibody (Bethyl Labs, Montgomery, TX; cat. #A-90-100A), polyclonal rabbit anti-mouse CD3 antibody (Abcam, Cambridge, MA; cat. #ab5690), or monoclonal rat anti-mouse CD45R antibody (Becton Dickinson, Franklin Lakes, NJ; cat. #550286) at the MSU Investigative Histopathology Laboratory to detect total IgG, CD3^+^ T lymphocytes, and CD45R^+^ B lymphocytes, respectively. Slides were scanned with a Slideview VS200 research slide scanner (Olympus, Hicksville, NY). Semi-quantitative scores for IgG deposition in kidneys were assigned using the following scale: (0) no changes compared to VEH/CON mice, (1) minimal (<10% affected), (2) mild (11-25% affected), (3) moderate (26-50% affected), (4) marked (51-75% affected), or (5) severe (76-100% affected).

### High-throughput autoantibody profiling

IgG and IgM AAbs were profiled in plasma (Study 2) by OmicsArray™ Systemic Autoimmune-associated Antigen Array (Genecopoiea Inc., Rockville, MD; cat. #PA001). All plasma samples within experimental groups were pooled prior to analysis. Briefly, plasma samples were incubated on microscope slides with 120 purified antigens adhered to nitrocellulose filters. One identical OmicsArray panel was reserved for a PBS negative sample control. After incubation, all slides were washed and incubated with Cy3-labeled anti-mouse IgG and Cy5-labeled anti-mouse IgM secondary antibodies. Slides were washed and fluorescent signals (532 nm for Cy3/IgG, 635 nm for Cy5/IgM) were detected using a GenePix^®^ 4400B microarray scanner (Molecular Devices, San Jose, CA), and GenePix^®^ 7.0 software (Molecular Devices) was used to determine fluorescent signal intensity values. Antibody scores (Ab-scores) for all AAbs were calculated using normalized signal intensity (NSI) and signal-to-noise ratio (SNR) values using the following formula:


$$Ab-score=\log_{2}(\text{NSI}\times \text{SNR}+1)$$

### Kidney mRNA expression

Total RNA from kidneys was extracted using TissueLyser II (Qiagen, Germantown, MD) and a RNeasy Mini Kit (Qiagen; cat. #74104) according to the manufacturer’s instructions. Isolated RNA was reconstituted in RNase-free water and quantified using a Nanodrop ND-1000 spectrophotometer (Thermo Fisher Scientific, Waltham, MA). cDNA was prepared from isolated RNA at a concentration of 100 ng/µl using a High-Capacity cDNA Reverse Transcriptase Kit (Thermo Fisher Scientific, Waltham, MA). Taqman assays were run with technical triplicates using a Smart Chip Real-Time PCR System at the MSU Genomics Core to assess interleukin (*Il1a, Il1b, Il2, Il6, Il17a, Il18*), chemokine (*Ccl2*, *Ccl7*, *Ccl12*, *Cxcl9*, *Cxcl10*, *Cxcl13*), inflammation/autoimmunity (*C1qa*, *C3*, *Casp1*, *Casp4*, *Icam1*, *Ifng*, *Lbp*, *Nfkb1*, *Nlrp3*, *Nos2*, *Pparg*, *Tlr4*, *Tlr9*, *Tnfa*, *Tnfsf13b*), type I interferon (IFN)-related (*Ifi44*, *Irf7*, *Isg15*, *Nlrc5*, *Oas2*), eicosanoid-related (*Alox15*, *Cyp2c44*, *Cyp2j6*, *Cyp2j9*, *Cyp2j11*, *Ephx1*, *Ephx2*, *Pla2g4c*, *Ptgs2*), kidney injury (*Ankrd1*, *Cd14*, *Havcr1*, *Tgfbr1*), oxidative stress-related (*Hmox*, *Ncf1*, *Nqo1*, *Sod2*), and housekeeping (*Actb*, *Gusb*) gene expression. Raw Ct values for each gene were converted to ΔCt values by subtracting the average Ct of the housekeeping genes from the Ct of the specified gene, and ΔΔCt values for each gene were calculated relative to the VEH/CON group by subtracting the average VEH/CON ΔCt value from individual ΔCt values within all experimental groups. The ΔΔCt values for each gene were then converted to relative copy number (RCN) values using the following equation (68):


$$RCN=2^{-\Delta \Delta Ct}$$

### Data analysis and statistics

All statistical analyses were conducted using GraphPad Prism Version 9 (GraphPad Software, San Diego, CA, www.graphpad.com). Outliers were identified using Grubb’s outlier test (*Q* = 1%), and normality was assessed using the Shapiro-Wilk test (*p* < 0.01). Quantitative data that failed to meet the assumption of normality and semi-quantitative data were analyzed by the Kruskal-Wallis nonparametric test followed by Dunn’s *post-hoc* test. The Brown-Forsythe test (*p* < 0.01) was used to test the assumption of equal variances across treatment groups. Normal data with unequal variances were analyzed using the Brown-Forsythe/Welch analysis of variance (ANOVA) followed by Dunnett’s T3 *post-hoc* test. Normal data that met the assumption of equal variance were analyzed by standard one-way ANOVA followed by Tukey’s *post-hoc* test. Data are presented as mean ± standard error of the mean (SEM), with a p-value < 0.05 considered statistically significant.

## Results

### Treatment with R-LPS but not S-LPS induces GN

**Figure 2:**
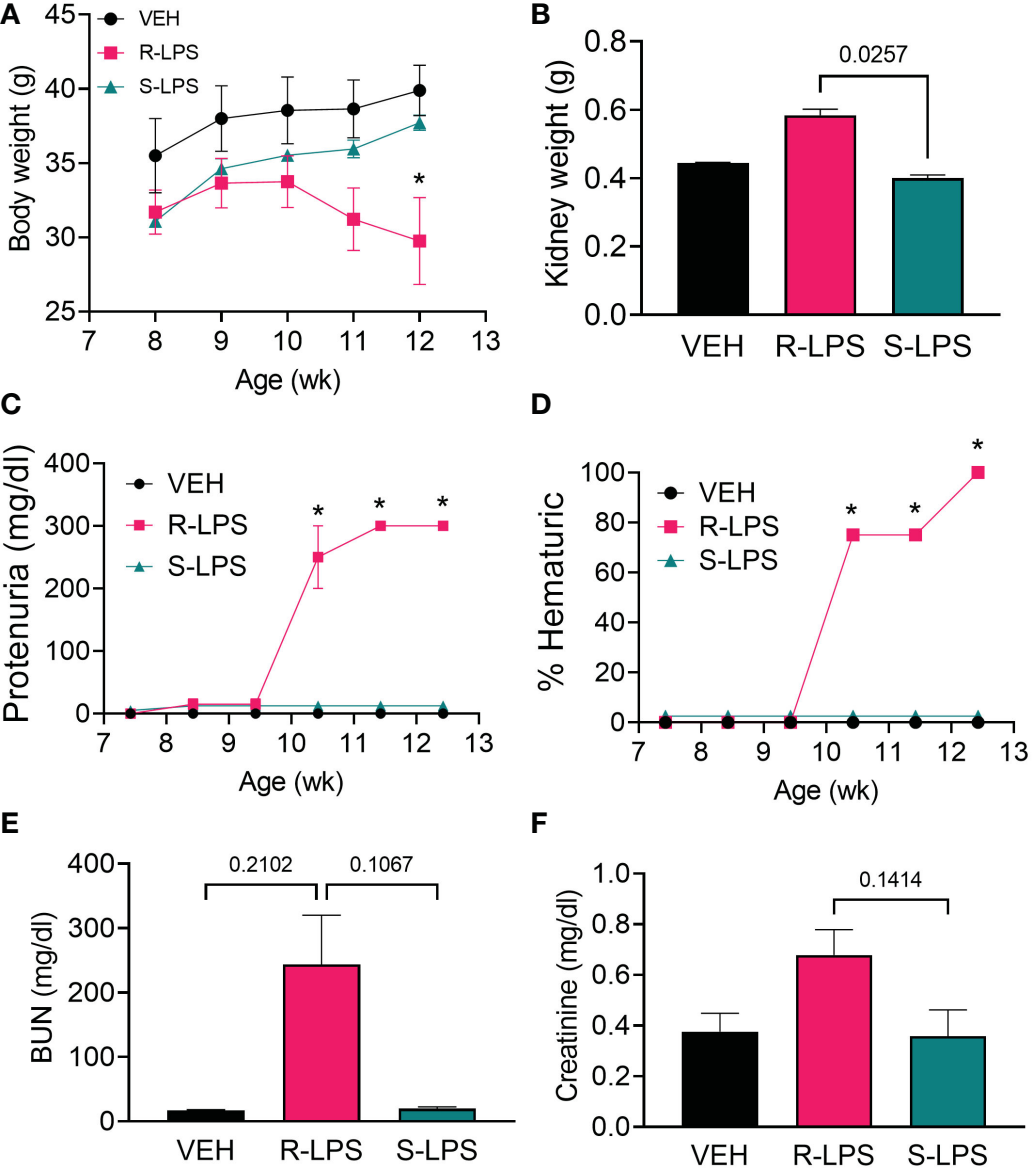
R-LPS but not S-LPS suppresses body weight gain, induces kidney enlargement, proteinuria, hematuria, and elevates BUN and creatinine in blood. **(A)** Mice were weighed weekly, concurrently with the first LPS injection of the wk. Data are presented as mean ± SEM. **(B)** Combined weight of left and right kidneys were measured after 5 wk of i.p. LPS injections. Data are presented as mean ± SEM. R-LPS, but not S-LPS, elicits robust proteinuria **(C)** and hematuria **(D)** after 3 wk of intraperitoneal (i.p.) LPS injections. Animals were monitored weekly for development of proteinuria (≥300 mg/dl urinary protein) and hematuria (>0 cells/µl urine) using clinical dipsticks. Blood urea nitrogen **(E)** and creatinine **(F)** were measured in plasma after 5 wk of i.p. LPS injections. BUN and creatinine data are presented as mean ± SEM. For **(A, C, D)**, *p<0.05 indicates statistical significance for R-LPS vs. VEH and R-LPS vs. S-LPS. For **(B, E, F)**, values of p<0.25 are shown, with p<0.05 considered statistically significant.

In Study 1 (**Figure 1A**), no significant differences in weight change among experimental groups were observed from 8 to 10 wk of age (**Figure 2A**). Beginning at age 10 wk, mice in the R-LPS group began losing weight while the weights of animals in the VEH and S-LPS groups steadily increased. The average combined kidney weight (sum of left kidney and right kidney) approximated to 0.45 g and 0.40 g within the VEH and S-LPS groups, respectively, at experiment termination (age 13 wk), whereas combined kidney weight the in R-LPS group increased to 0.58 g (**Figure 2B**). In line with these findings, mice in the R-LPS group alone began exhibiting proteinuria (**Figure 2C**) and hematuria (**Figure 2D**) after age 10 wk, whereas animals in the VEH and S-LPS groups displayed neither proteinuria nor hematuria at any point during the study. At age 13 wk, trends toward increased blood urea nitrogen (BUN) (**Figure 2E**) and plasma creatinine (**Figure 2F**) were observed in the R-LPS group compared to the VEH and S-LPS groups.

**Figure 3:**
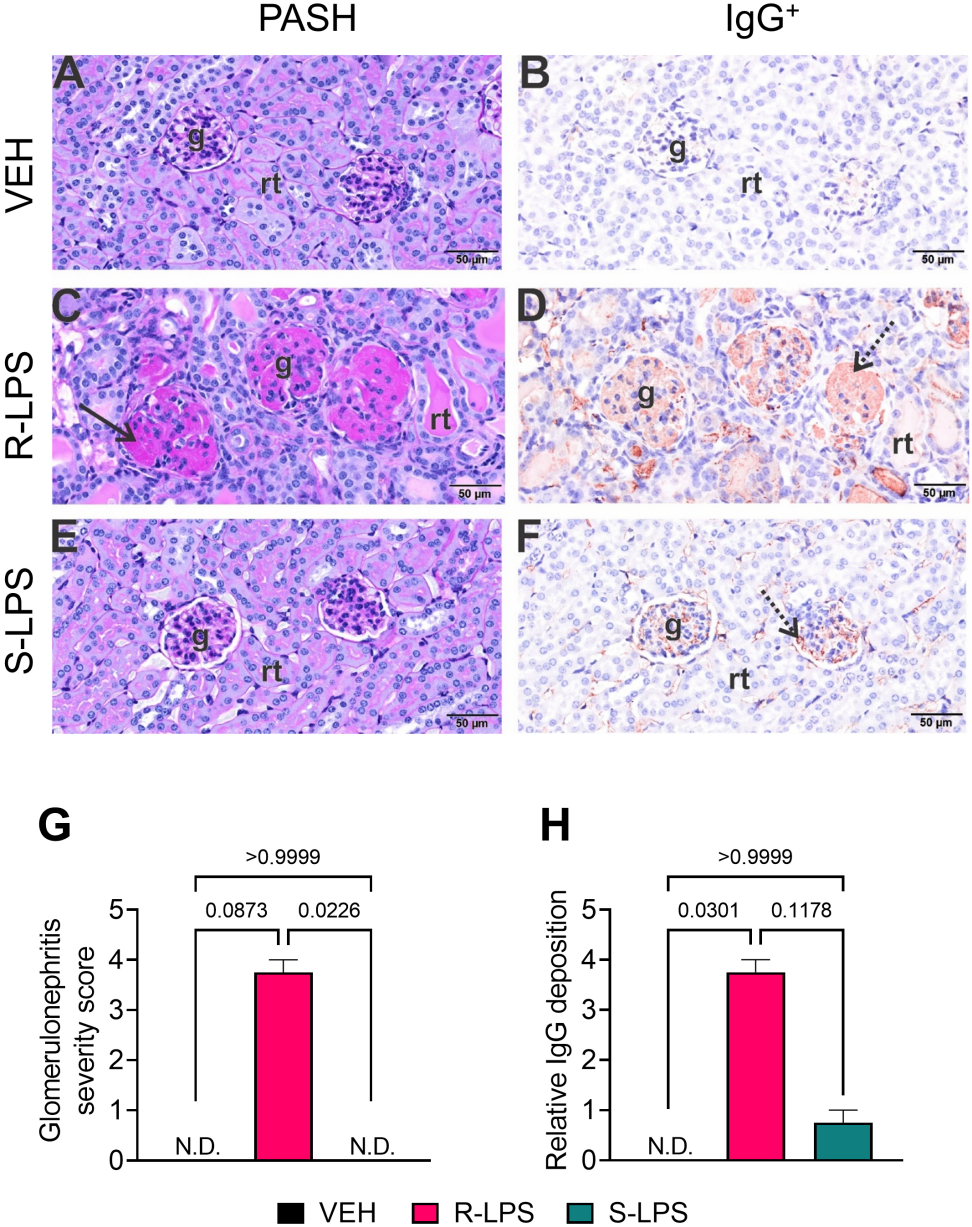
R-LPS but not S-LPS induces glomerulonephritis. Light photomicrographs of glomeruli (g) and renal tubules (rt) in the cortex of kidneys from vehicle-treated control mice **(A, B)**, rough (R) LPS-treated mice **(C, D)**, and smooth LPS-treated mice **(E, F)**. Renal tissues were histochemically stained with periodic acid Schiff and hematoxylin (PASH) **(A, C, E)** and immunohistochemically stained for IgG protein and counterstained with hematoxylin **(B, D, F)**. Hypertrophic glomeruli with markedly thickened periodic acid fast-stained medullary membranes (solid arrow), hyalinized proteinaceous material in renal tubular lumens, and mild lymphoplasmacytic infiltrate in cortical interstitial tissue of R-LPS-treated mice **(C)**. Correspondingly, immunohistochemically stained IgG in glomeruli (stippled arrow), renal tubular lumens and blood vessel lumens **(D)**. Minimal to no PAS+ medullary membrane thickening in glomeruli of S-LPS-treated mice **(E)** with minimal IgG+ medullary material **(F)**. Semi-quantitative scores for **(G)** glomerulonephritis severity and **(H)** IgG deposition. Scoring was as follows: 0—no significant finding, 1—minimal, 2—mild, 3—moderate, 4—marked, 5—severe. See text for detailed criteria used in severity scoring. Data are presented as mean ± SEM (*n* = 2-4). Values of p<0.1 are shown, with p<0.05 considered statistically significant. g, glomerulus; rt, renal tubule; N.D., not determined.

Examination of periodic acid Schiff and hematoxylin (PASH)-stained renal sections and subsequent semi-quantitative scoring revealed minimal to no PAS+ medullary membrane thickening in glomeruli of VEH- and S-LPS-treated mice (**Figures 3A, E, G**). In contrast, kidneys of R-LPS-treated mice contained markedly hypertrophic glomeruli with thickened periodic acid fast-stained medullary membranes, hyalinized proteinaceous material in renal tubular lumens, and mild lymphoplasmacytic infiltrate in cortical interstitial tissue, all of which were indicative of GN (**Figures 3C, G**). Consistent with these findings, immunohistochemical staining indicated that R-LPS but not S-LPS induced glomerular deposition of IgG (**Figures 3B, D, F, H**). In further congruence with histopathology findings, renal tissue from VEH-injected mice exhibited no significant influx of CD45R^+^ B lymphocytes (**Supplementary Figure 1A**) and minimal influx of CD3^+^ T lymphocytes (**Supplementary Figure 1B**). On the other hand, R-LPS-injected mice demonstrated a moderate increase in renal CD45R^+^ lymphoid cell infiltration (**Supplementary Figure 1C**) and a marked increase in CD3^+^ lymphoid cell infiltration (**Supplementary Figure 1D**), while kidney tissues from S-LPS- injected mice resembled those from VEH-injected mice (**Supplementary Figures 1E, F**). CD45R^+^ and CD3^+^ lymphocytes did not localize to any specific region in the kidney. Altogether, blood and urine analyses, histopathology, and immunohistochemistry indicated R-LPS but not S-LPS induced robust GN.

### R-LPS but not S-LPS elicits lymphoid cell accumulation in spleen and liver

**Figure 4:**
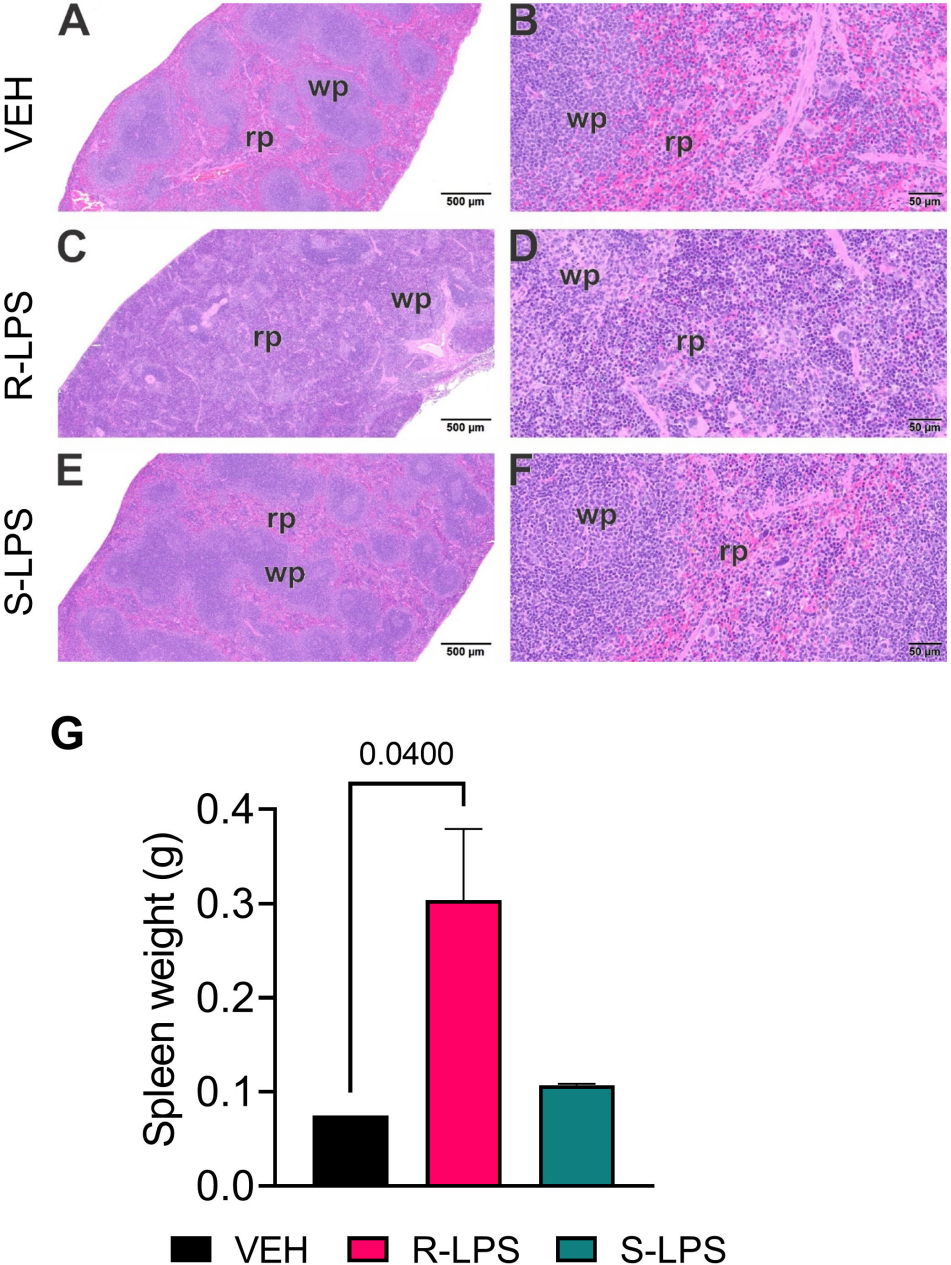
R-LPS but not S-LPS induces lymphoid cell hyperplasia and enlargement of spleen. Light photomicrographs of transverse hematoxylin and eosin-stained tissues from the body of spleens in vehicle (VEH)-treated control mice **(A, B)**, rough (R)-LPS-treated mice **(C, D)**, and smooth-(S) LPS-treated mice **(E, F)**. **(A, C, E)** taken at low magnification and **(B, D, F)** taken at higher magnification. Splenic tissue from R-LPS mice **(B, D)** have lymphoid cell hyperplasia in white pulp (wp) with correspondingly lesser red pulp (rp). No histopathology in spleens of S-LPS-treated mice **(E, F)** that were histologically similar to vehicle-treated control mice **(A, B)**. R-LPS but not S-LPS contributes to larger spleen weight after 5 wk of i.p. injections **(G)**.

**Figure 5:**
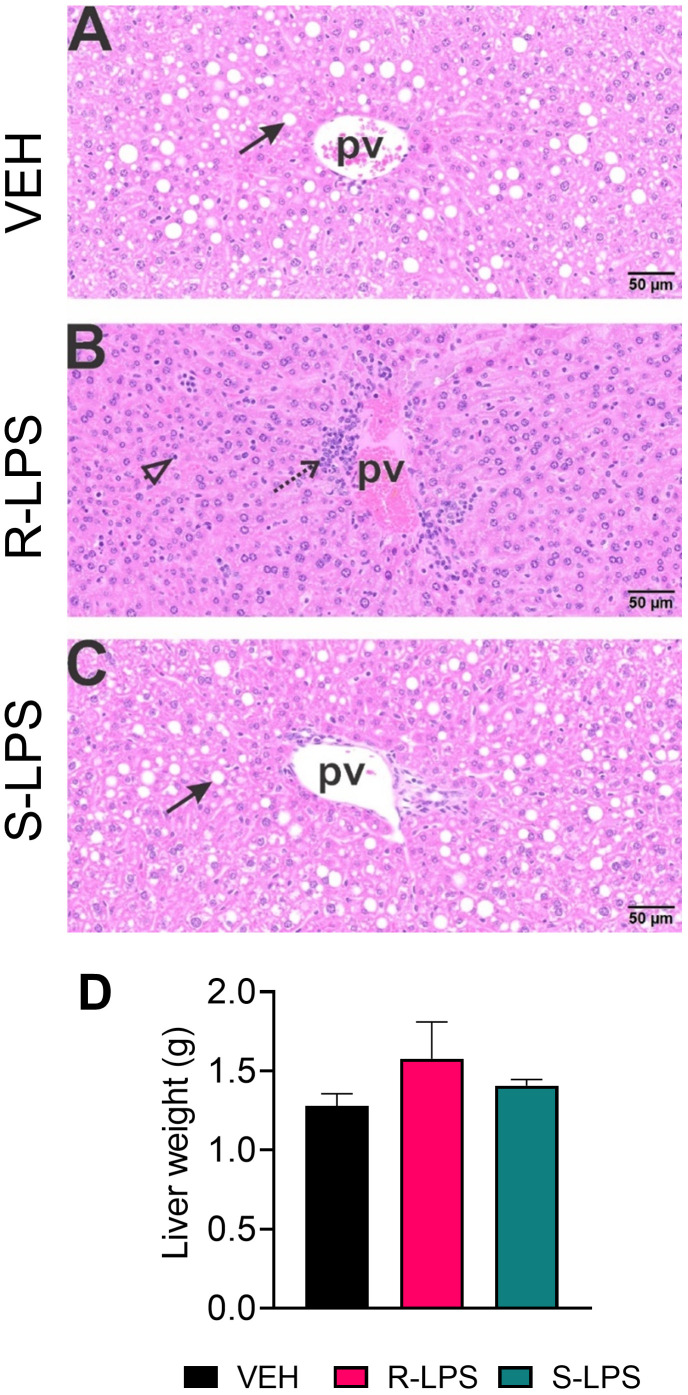
R-LPS but not S-LPS induces lymphoid cell accumulation and reduces vacuolization in liver. Light photomicrographs of hematoxylin and eosin-stained hepatic tissue from **(A)** vehicle (VEH)-treated control, **(B)** rough (R)-LPS-treated mice, and **(C)** smooth (S)-LPS-treated mice. Periportal large and small hepatocellular vacuoles resembling fatty liver histopathology (steatosis) in vehicle control mouse (arrow) **(A)**. Periportal interstitial lymphoid cell accumulation in rough-LPS-treated mouse **(B)** without hepatocellular vacuolization. Histology of liver tissue from smooth-LPS mouse **(C)** resembles that of vehicle control mouse **(A)**. R-LPS and S-LPS effects on liver weight are negligible **(D)**. Solid arrow, hepatocellular lipid vacuoles; stippled arrow, periportal cellular inflammation (predominantly mononuclear cells); open arrowhead, mononuclear cells in hepatic sinusoids.

Spleen and liver tissue sections were also histologically evaluated after Study 1 termination (**Figures 4**, **5**). No histopathology was evident in spleens of VEH-treated control mice (**Figures 4A, B**) that was histologically similar to S-LPS-treated mice (**Figures 4E, F**). Splenic tissue from R-LPS mice (**Figures 4C, D**) had lymphoid cell hyperplasia in white pulp with correspondingly lesser red pulp. Consistent with the expansion of white pulp, the R-LPS group showed a marked average weight increase at 0.30 g compared to the VEH and S-LPS groups at 0.08 g and 0.11g, respectively (**Figure 4G**). Histologic assessment of liver showed periportal large and small hepatocellular vacuoles resembling fatty liver histopathology (steatosis) in VEH-treated control mice (**Figure 5A**). There was marked periportal interstitial lymphoid cell accumulation in R-LPS-treated mice without hepatocellular vacuolization (**Figure 5B**). Histology of liver tissue from S-LPS mice (**Figure 5C**) resembled that of VEH/CON mice. Average liver weights did not significantly change with either R-LPS (1.58 g) or S-LPS (1.41 g) compared to the VEH group (1.28 g) (**Figure 5D**). Accordingly, R-LPS but not S-LPS caused enlargement and lymphoid cell expansion in the spleen as well as modest lymphoid cell recruitment in the liver.

### DHA supplementation selectively modulates red blood cell PUFA profile

**Figure 6:**
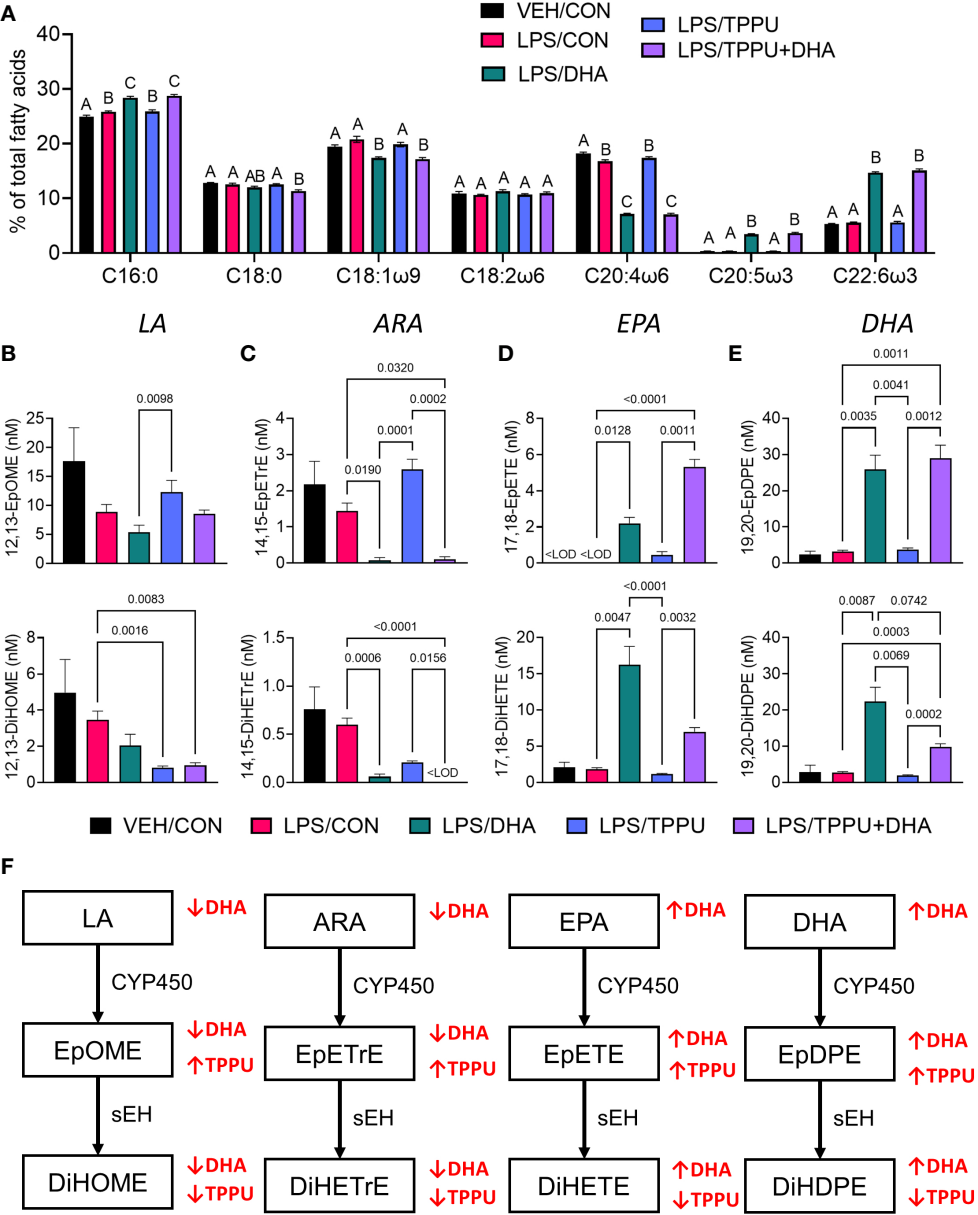
Supplementation with DHA and/or TPPU modulates polyunsaturated fatty acid (PUFA) and CYP450 metabolite profiles in red blood cell membranes and plasma. **(A)** DHA consumption elevates ω-3 PUFA DHA and EPA in red blood cell membrane at the expense of ω-6 PUFA arachidonic acid and ω-9 PUFA oleic acid. Major red blood cell fatty acids were compared across treatment groups by GLC and expressed as percent of total fatty acids. Different letters indicate statistically significant differences between treatment groups for individual fatty acids (p<0.05). C16:0, palmitic acid; C18:0, stearic acid; C18:1n9, oleic acid; C18:2n6, linoleic acid; C20:4n6, arachidonic acid; C20:5n3, eicosapentaenoic acid (EPA); C22:6n3, docosahexaenoic acid (DHA). **(B–D)** Following sacrifice, plasma was isolated and selected **(B)** LA metabolites (i.e., 12,13-EpOME, 12,13-DiHOME), **(C)** ARA metabolites (i.e., 14,15-EpETrE, 14,15-DiHETrE), **(D)** EPA metabolites (i.e., 17,18-EpETE, 17,18-DiHETE), and **(E)** DHA metabolites (i.e., 19,20-EpDPE, 19,20-DiHDPE) were measured by LC-MS/MS. Data are presented as mean ± SEM (*n =* 6-8). Values of p<0.1 are shown, with p<0.05 considered statistically significant.<LOD = below limit of detection. **(F)** Depiction of pathways to major epoxy- and dihydroxy-fatty acid metabolites derived from ω-6 and ω-3 PUFAs. ω-6 and ω-3 PUFA are substrates of CYP450 monooxygenases, which produce ω-6 and ω-3 epoxy-fatty acids, respectively. The resulting epoxy-fatty acids then are converted to their corresponding ω-6/ω-3 dihydroxy-fatty acids by soluble epoxide hydrolase (sEH). Red arrows indicate how fatty acid and metabolite profiles are affected by LA, linoleic acid; DGLA, dihomo-gamma-linolenic acid; ARA, arachidonic acid; EpOME, epoxyoctadecenoic acid; EED, epoxyeicosadienoic acid; DHED, dihydroxyeicosadienoic acid; ARA, arachidonic acid; EpETrE, epoxyeicosatrienoic acid; DiHETrE, dihydroxyeicosatrienoic acid; ALA, alpha-linolenic acid; EpODE, epoxyoctadecadienoic acid; DiHODE, dihydroxyoctadecadienoic acid; EPA, eicosapentaenoic acid; EpETE, epoxyeicosatetraenoic acid; DiHETE, dihydroxyeicosatetraenoic acid; DHA, docosahexaenoic acid; EpDPE, epoxydocosapentaenoic acid; DiHDPE, dihydroxydocosapentaenoic acid.

In Study 2, we compared the effects of i.p. injection of R-LPS on GN and related endpoints in mice fed control CON, DHA, TPPU, and TPPU+DHA diets (**Figure 1B**). When total red blood cell fatty acids including saturated fatty acids (SFAs), monounsaturated fatty acids (MUFAs), ω-6 PUFAs, and ω-3 PUFAs were determined by GLC, the seven most abundant fatty acids were palmitic acid (PA, C16:0), stearic acid (SA, C18:0), oleic acid (OA, C18:1ω9), linoleic acid (LA, C18:2ω6), arachidonic acid (ARA, C20:4ω6), EPA (C20:5ω3), and DHA (C22:6ω3) (**Table 2** and **Figure 6A**). LPS treatment had no effect on fatty acid profiles of CON-fed mice. Consistent with prior findings (63), we found that substituting high-oleic safflower oil with DHA-rich algal oil in the AIN-93G diet increased incorporation of DHA and EPA into the red blood cell membrane, at the expense of ARA and OA. There was also a slight increase in membrane LA while SA slightly decreased with dietary DHA incorporation. The ω-3 index, or measure of EPA and DHA in relation to total red blood cell fatty acids (69), was elevated in mice that received either DHA or TPPU+DHA diet. TPPU administration alone had no significant effect on total membrane SFAs, MUFA, and PUFAs. Overall, feeding DHA elevated ω-3 PUFAs and decreased total MUFAs and ω-6 PUFAs.

**Table 2:** Red blood cell fatty acid content as determined by GLC.

Common name	Chemical formula	VEH/CON	LPS/CON	LPS/DHA	LPS/TPPU	LPS/TPPU+DHA
Myristic	C14:0	0.18 ± 0.00^A^	0.16 ± 0.00^A^	0.36 ± 0.00^BC^	0.18 ± 0.00^A^	0.36 ± 0.00^BC^
Palmitic	C16:0	24.95 ± 0.01^A^	25.81 ± 0.00^A^	28.43 ± 0.01^C^	25.87 ± 0.01^A^	28.76 ± 0.01^C^
Palmitolaidic	C16:1ω7t	0.05 ± 0.00^A^	0.04 ± 0.00^A^	0.03 ± 0.00^B^	0.04 ± 0.00^AB^	0.04 ± 0.00^AB^
Palmitoleic	C16:1ω7	0.89 ± 0.00^A^	0.74 ± 0.00^A^	0.79 ± 0.00^A^	0.75 ± 0.00^A^	0.76 ± 0.00^A^
Stearic	C18:0	12.78 ± 0.00^A^	12.56 ± 0.01^AB^	11.98 ± 0.01^A^	12.57 ± 0.00^A^	11.35 ± 0.00^BC^
Elaidic	C18:1t	0.15 ± 0.00^AB^	0.15 ± 0.00^B^	0.12 ± 0.00^C^	0.15 ± 0.00^AB^	0.12 ± 0.00^C^
Oleic	C18:1ω9	19.48 ± 0.01^AB^	20.78 ± 0.02^B^	17.92 ± 0.00^AC^	19.84 ± 0.01^AB^	17.19 ± 0.01^C^
Linoelaidic	C18:2ω6t	0.09 ± 0.00^A^	0.10 ± 0.00^A^	0.05 ± 0.00^C^	0.08 ± 0.00^AB^	0.05 ± 0.00^C^
Linoleic	C18:2ω6	10.89 ± 0.01^AB^	10.60 ± 0.00^A^	11.30 ± 0.01^AB^	10.67 ± 0.01^AB^	10.95 ± 0.01^AB^
Alpha-linolenic	C18:3ω3	0.05 ± 0.00^A^	0.05 ± 0.00^A^	0.06 ± 0.00^A^	0.04 ± 0.00^A^	0.06 ± 0.00^A^
Gamma-linolenic	C18:3ω6	0.07 ± 0.00^A^	0.07 ± 0.00^A^	0.04 ± 0.00^B^	0.07 ± 0.00^A^	0.04 ± 0.00^B^
Arachidic	C20:0	0.18 ± 0.00^A^	0.15 ± 0.00^AB^	0.13 ± 0.00^B^	0.15 ± 0.00^AB^	0.16 ± 0.00^A^
Eicosenoic	C20:1ω9	0.38 ± 0.00^A^	0.39 ± 0.00^A^	0.25 ± 0.00^CD^	0.40 ± 0.00^A^	0.23 ± 0.00^D^
Eicosadienoic	C20:2ω6	0.31 ± 0.00^A^	0.32 ± 0.00^A^	0.24 ± 0.00^CD^	0.32 ± 0.00^A^	0.23 ± 0.00^D^
Dihomo-γ-linolenic	C20:3ω6	1.34 ± 0.00^A^	1.30 ± 0.00^A^	0.94 ± 0.00^CD^	1.32 ± 0.00^A^	0.90 ± 0.00^D^
Arachidonic	C20:4ω6	18.18 ± 0.01^A^	16.80 ± 0.01^AB^	7.16 ± 0.00^C^	17.42 ± 0.01^AC^	7.04 ± 0.01^D^
Behenic	C22:0	0.63 ± 0.00^A^	0.45 ± 0.00^AB^	0.23 ± 0.00^BC^	0.48 ± 0.00^AB^	0.31 ± 0.00^BC^
Eicosapentaenoic	C20:5ω3	0.36 ± 0.00^A^	0.37 ± 0.00^A^	3.41 ± 0.00^B^	0.35 ± 0.00^A^	3.64 ± 0.00^B^
Docosapentaenoic ω-3	C22:5ω3	0.66 ± 0.00^A^	0.70 ± 0.00^A^	1.04 ± 0.00^B^	0.67 ± 0.00^A^	1.02 ± 0.00^B^
Docosapentaenoic ω-6	C22:5ω6	0.74 ± 0.00^A^	0.69 ± 0.00^AB^	0.20 ± 0.00^C^	0.72 ± 0.00^A^	0.24 ± 0.00^BC^
Docosahexaenoic	C22:6ω3	5.33 ± 0.00^A^	5.55 ± 0.00^AB^	14.69 ± 0.00^C^	5.63 ± 0.00^AB^	15.11 ± 0.01^C^
Lignoceric	C24:0	0.43 ± 0.00^A^	0.38 ± 0.00^AB^	0.30 ± 0.00^BC^	0.40 ± 0.00^AC^	0.49 ± 0.00^A^
Nervonic	C24:1ω9	0.41 ± 0.00^AB^	0.35 ± .000^ABC^	0.26 ± 0.00^C^	0.39 ± 0.00^AB^	0.38 ± 0.00^A^
Σ SFA		39.15 ± 0.01^A^	39.51 ± 0.01^A^	41.44 ± 0.01^B^	39.64 ± 0.01^AB^	41.42 ± 0.01^B^
Σ MUFA		21.35 ± 0.01^AB^	22.46 ± 0.02^A^	18.87 ± 0.01^B^	21.55 ± 0.01^AB^	18.72 ± 0.01^C^
Σ ω-3 PUFA		6.40 ± 0.00^A^	6.67 ± 0.00^AB^	19.19 ± 0.01^C^	6.70 ± 0.01^AB^	19.83 ± 0.01^C^
Σ ω-6 PUFA		33.10 ± 0.01^A^	31.37 ± 0.01^B^	20.50 ± 0.01^D^	32.11 ± 0.01^AB^	20.02 ± 0.01^D^
ω-3 Index		5.69 ± 0.00^A^	5.92 ± 0.00^AB^	18.10 ± 0.01^C^	5.98 ± 0.00^AB^	18.75 ± 0.01^C^

Data are presented as percent of total fatty acids (mean ± SEM, n = 8/gp) as measured by GLC. Differences between experimental groups were compared by ordinary one-way ANOVA followed by Tukey’s post-hoc test. Nonparametric versions of these tests were used when applicable. Unique letters indicate significant differences between groups (p<0.05).

### Consumption of DHA- and/or TPPU-amended diets selectively skew plasma CYP450 metabolite profiles

**Figure 7:**
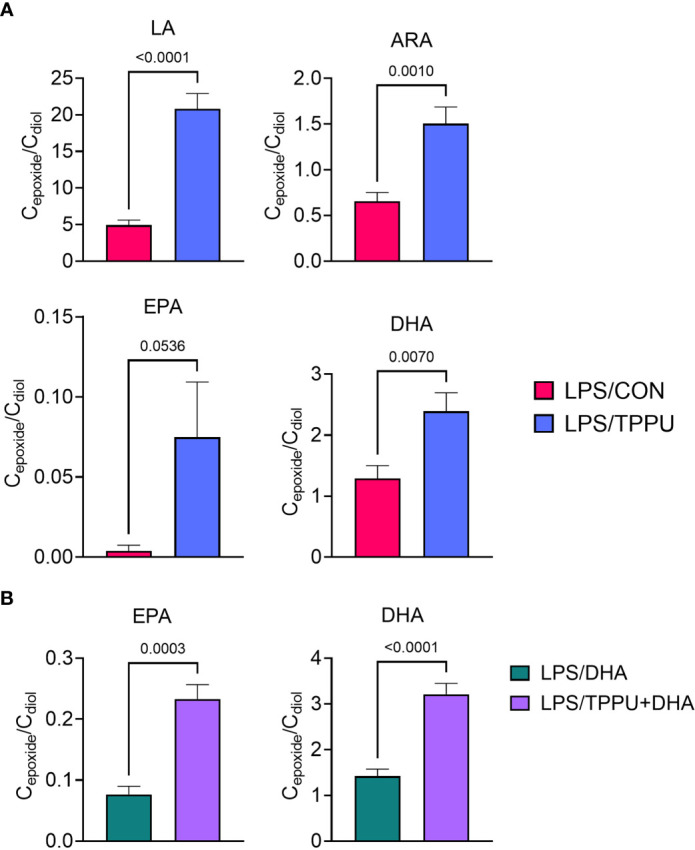
Supplementation with DHA and/or TPPU modulates plasma epoxide/diol metabolite ratios in LPS-injected NZBWF1 mice. **(A)** TPPU consumption significantly shifts epoxide/diol ratios for metabolites derived from LA, ARA, EPA, and DHA. Ratios between sums of all EpFAs and sums of all DiHFAs from each PUFA precursor are shown. **(B)** DHA supplementation elevates epoxide/diol ratios for EPA-derived metabolites, and combination of DHA+TPPU further shifts epoxide/diol ratios for EPA- and DHA-derived metabolites. Ratios between sums of all EpFAs and sums of all DiHFAs from each PUFA precursor are shown. Data are presented as mean ± SEM (*n =* 8). Values of p<0.1 are shown, with p<0.05 considered statistically significant.

Omega-6 and ω-3 PUFAs act as substrates for CYP450 monooxygenases, which convert the parent PUFA into epoxy-fatty acids (EpFAs). In turn, EpFAs act as substrates for sEH, which converts EpFAs into their corresponding vicinal diols, dihydroxy-fatty acids (DiHFAs). Inclusion of TPPU in experimental diets resulted in presence of 5 to 6 µM of the drug in plasma (**Supplementary Figure 2**), which is consistent with the TPPU blood concentration obtained from efficacious doses (3 mg/kg/day) in other preclinical studies without reported side effects (41, 70–72). We assessed the impacts of DHA and TPPU on plasma levels of EpFAs, DiHFAs, and other PUFA-derived oxylipins using a comprehensive LC-MS/MS oxylipin panel (**Supplementary Table 6**). Prominent metabolites included ones derived from LA (i.e., 12,13-EpOME and 12,13-DiHOME) (**Figure 6B**), ARA (i.e., 14,15-EpETrE and 14,15-DiHETrE) (**Figure 6C**), EPA (i.e., 17,18-EpETE and 17,18-DiHETE**)** (**Figure 6D**), and DHA (i.e., 19,20-EpDPE and 19,20-DiHDPE) (**Figure 6E**). No significant changes were observed between VEH/CON and LPS/CON mice. Consistent with our total red blood cell fatty acid data (**Figure 6A**) and prior reports (73–75), we found that substituting high-oleic safflower oil with DHA-rich algal oil elicited decreases in plasma LA- and ARA-derived EpFAs and DiHFAs and corresponding increases in plasma EPA- and DHA-derived EpFAs and DiHFAs (**Figure 6F**). Increases in DHA-derived metabolites were much more pronounced than those of EPA-derived metabolites. In addition, mice in the LPS/TPPU group exhibited modest increases in LA-, ARA-, EPA- and DHA-derived EpFAs compared to the LPS/CON group, whereas LPS/TPPU displayed a modest decrease in 14,15-DiHETrE but not 17,18-DiHETE and 19,20-DiHDPE. Furthermore, the LPS/TPPU+DHA group displayed modest increases in 14,15-EpETrE and 17,18-EpETE compared to the LPS/TPPU group, although these changes were not statistically significant with the LPS/CON and LPS/DHA groups; 19,20-EpDPE levels were not significantly affected by TPPU. Furthermore, TPPU+DHA co-treatment increased 17,18-DiHETE and 19,20-DiHDPE relative to the TPPU group and caused modest, but not significant, decreases in 14,15-DiHETrE, 17,18-DiHETrE, and 19,20-DiHDPE compared to CON- or DHA-fed mice. In summary, the LPS/TPPU group exhibited significant increases in epoxide/diol ratios for LA-, ARA-, EPA-, and DHA-derived metabolites compared to the LPS/CON group (**Figure 7A** and **Table 3**), and the LPS/TPPU+DHA group exhibited significant increases epoxide/diol ratios in EPA- and DHA-derived metabolites compared to the LPS/DHA group (**Figure 7B** and **Table 3**).

**Table 3:** Plasma epoxide/diol metabolite ratios at necropsy as determined by LC-MS/MS.

Fatty acid precursor	VEH/CON	LPS/CON	LPS/DHA	LPS/TPPU	LPS/TPPU+DHA
LA	6.84 ± 0.67	4.92 ± 0.69^A^	6.29 ± 1.28^A^	20.83 ± 2.07^B^	15.37 ± 2.36^B^
ARA	0.72 ± 0.18	0.66 ± 0.09^AB^	0.40 ± 0.15^A^	1.50 ± 0.18^B^	2.10 ± 1.14^AB^
EPA	0.00 ± 0.00	0.00 ± 0.00^A^	0.08 ± 0.01^AB^	0.07 ± 0.03^A^	0.23 ± 0.02^B^
DHA	1.69 ± 0.72	1.29 ± 0.21^A^	1.42 ± 0.15^A^	2.39 ± 0.30^AB^	3.21 ± 0.24^B^
Σ ω-6 PUFA	1.80 ± 0.37	1.63 ± 0.15^A^	1.52 ± 0.23^A^	3.93 ± 0.63^B^	4.84 ± 0.89^B^
Σ ω-3 PUFA	0.13 ± 0.03	0.27 ± 0.04*^A^	0.54 ± 0.07^AB^	0.61 ± 0.09^AB^	1.00 ± 0.10^B^
Σ PUFA	0.94 ± 0.12	0.95 ± 0.10^AB^	0.64 ± 0.09^A^	2.08 ± 0.28^C^	1.34 ± 0.13^BC^

Data are presented as ratios between plasma epoxide metabolite concentrations (C_epoxide_) and plasma diol metabolite concentrations (C_diol_) (mean ± SEM, n = 8/gp). Epoxides and diols accounted for each fatty acid precursor included: LA) 9,10-EpOME, 9,10-DiHOME, 12,13-EpOME, 12,13-DiHOME; ARA) 5,6-EpETrE, 5,6-DiHETrE, 8,9-EpETrE, 8,9-DiHETrE, 11,12-EpETrE, 11,12-DiHETrE, 14,15-EpETrE, 14,15-DiHETrE; EPA) 5,6-EpETE, 5,6-DiHETE, 8,9-EpETE, 8,9-DiHETE, 11,12-EpETE, 11,12-DiHETE, 14,15-EpETE, 14,15-DiHETE, 17,18-EpETE, 17,18-DiHETE; DHA) 7,8-EpDPE, 7,8-DiHDPE, 10,11-EpDPE, 10,11-DiHDPE, 13,14-EpDPE, 13,14-DiHDPE, 16,17-EpDPE, 16,17-DiHDPE, 19,20-EpDPE, 19,20-DiHDPE. Ratios for total ω-6 PUFA were calculated from the sum of the LA- and ARA-derived epoxides and diols specified above. Ratios for total ω-3 PUFA were calculated from the sum of the EPA- and DHA-derived epoxides and diols specified above. Ratios for total PUFA were calculated from the sum of the ω-6- and ω-3-derived epoxides and diols described above. Differences between VEH/CON and LPS/CON groups were compared by Student’s t test. LPS/CON, LPS/DHA, LPS/TPPU, and LPS/TPPU+DHA groups were compared by ordinary one-way ANOVA followed by Tukey’s post-hoc test. Nonparametric versions of these tests were used when applicable. Asterisks (*) indicate significant differences between VEH/CON and LPS/CON groups (p<0.05). Unique letters indicate significant differences between LPS/CON, LPS/DHA, LPS/TPPU, and LPS/TPPU+DHA groups (p<0.05). LA, linoleic acid; ARA, arachidonic acid; EPA, eicosapentaenoic acid; DHA, docosahexaenoic acid; PUFA, polyunsaturated fatty acid.

### DHA and TPPU treatment alone suppress R-LPS-induced GN

**Figure 8:**
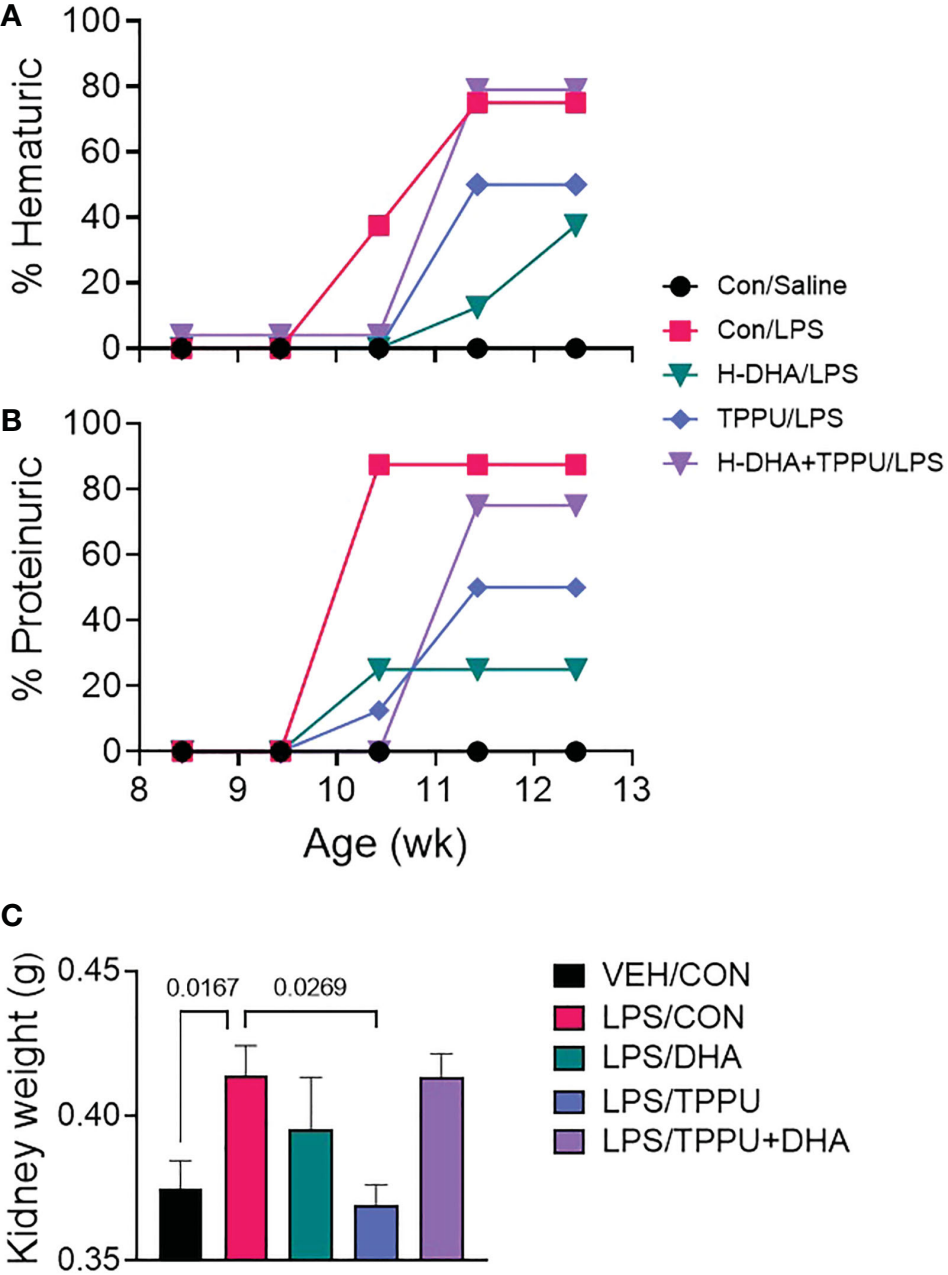
DHA alone and TPPU alone delay onset of R-LPS-induced hematuria and proteinuria but are antagonistic when delivered together. Animals were monitored weekly for development of **(A)** hematuria (>0 cells/µl urine) and **(B)** proteinuria (≥300 mg/dl urinary protein) using clinical dipsticks. **(C)** After 5 wk of biweekly i.p. LPS injections, mice were sacrificed, and both left and right kidneys were weighed before additional tissue processing. Data are presented as mean ± SEM. Statistically significant differences between VEH/CON and LPS/CON were assessed by Student’s t-test. LPS/DHA, LPS/TPPU, and LPS/TPPU+DHA were compared to LPS/CON using one-way ANOVA followed by Tukey’s *post-hoc* test. Values of p<0.1 are shown, with p<0.05 considered statistically significant.

For the duration of the study, mice in all experimental groups gained weight at similar rates, regardless of dietary intervention (**Supplementary Figure 3**). During the 5 wk of LPS injections, mice were assessed weekly for development of hematuria and proteinuria as indicators of GN (**Figures 8A, B**). Individuals in the VEH/CON group did not display proteinuria or hematuria. At 10 wk of age (after 6 injections), mice in the LPS/CON group began developing hematuria (**Figure 8A**). At age 11 wk (after 8 injections), mice in the LPS/DHA, LPS/TPPU, and LPS/TPPU+DHA experimental groups began developing hematuria. After the final injection, 75% of animals in the LPS/CON and LPS/TPPU+DHA groups displayed hematuria, 50% of animals in the LPS/TPPU group displayed hematuria, and 38% of animals in the LPS/DHA group displayed hematuria. In similar fashion, mice in the LPS/CON, LPS/DHA, and LPS/TPPU groups began developing proteinuria at age 10 wk (after 6 injections), and mice in the LPS/TPPU+DHA group began developing proteinuria at 11 wk of age (after 8 injections) (**Figure 8B**). After the final injection, proteinuria was evident in 87.5% of the LPS/CON group, 75% of the LPS/TPPU+DHA group, 50% of the LPS/TPPU group, and 38% of the LPS/DHA group. Consistent with GN, the average combined kidney weight was significantly elevated in the LPS/CON group compared to the VEH/CON group (**Figure 8C**). The only group that demonstrated a significant decrease in kidney weight compared to the LPS/CON group was the LPS/TPPU group, while no significant differences were observed for the other groups.

**Figure 9:**
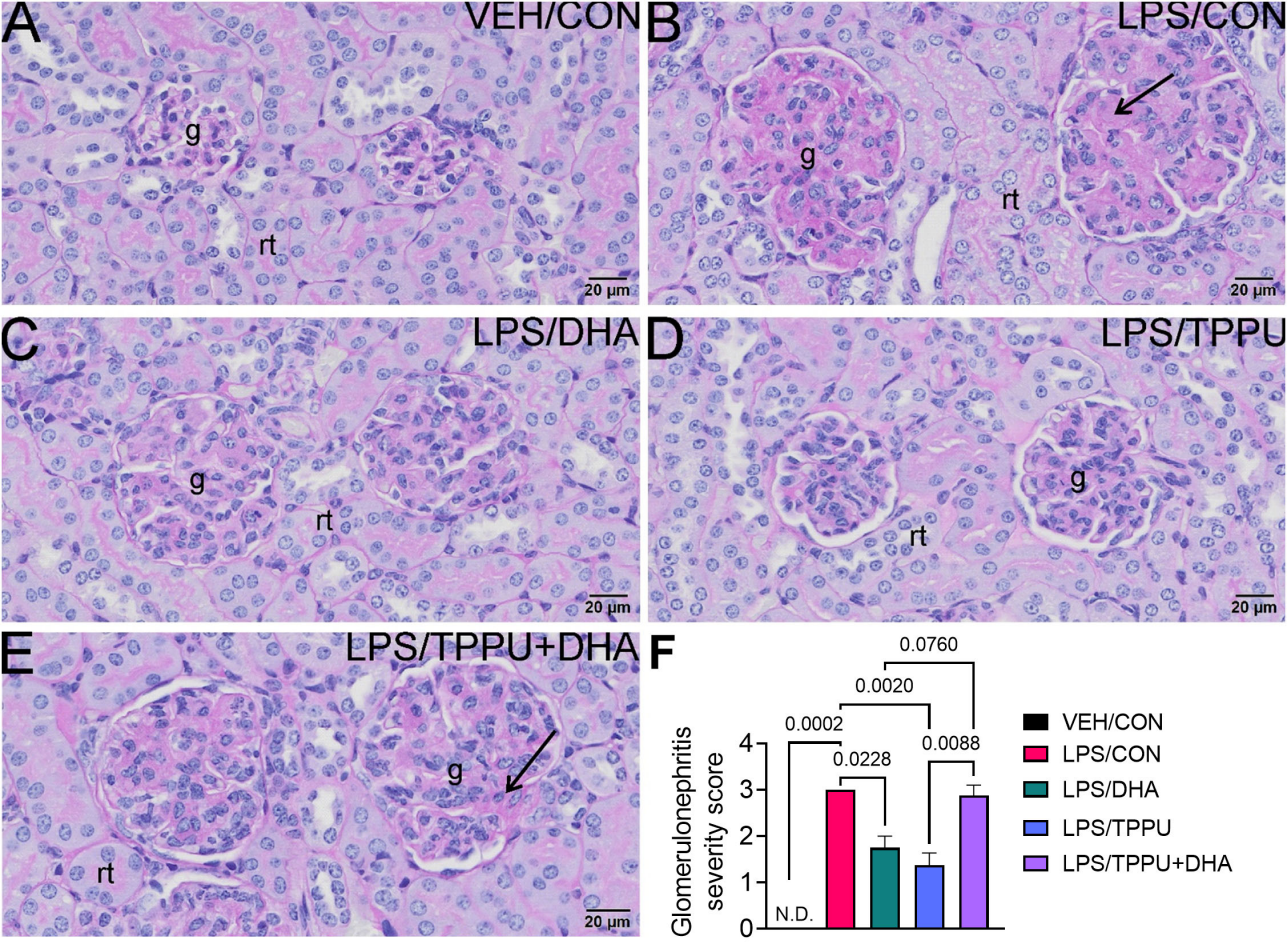
DHA alone and TPPU alone suppress R-LPS-induced glomerulonephritis but are antagonistic when delivered together. Light photomicrographs of periodic acid Schiff and hematoxylin (PASH)-stained cortical kidney tissue from **(A)** vehicle (VEH)-treated/control diet (CON) mouse, **(B)** rough LPS-treated/CON mouse, **(C)** LPS/DHA mouse, **(D)** LPS/TPPU mouse, and **(E)** LPS/TPPU+DHA mouse. Markedly hypertrophic and hypercellular glomeruli (g) with thickened medullary membranes in kidneys of LPS/CON mice **(B)** and LPS/TPPU+DHA mice **(E)**. Less glomerular histopathology in kidneys of LPS/DHA mice **(C)** and LPS/TPPU mice **(D)**. **(F)** Semi-quantitative scores for glomerulonephritis severity. Scoring was as follows: 0—no significant finding, 1—minimal, 2—mild, 3—moderate, 4—marked, 5—severe. Data are presented as mean ± SEM (*n =* 8). Values of p<0.1 are shown, with p<0.05 considered statistically significant. g, glomerulus; rt, renal tubule; N.D., not determined.

**Figure 10:**
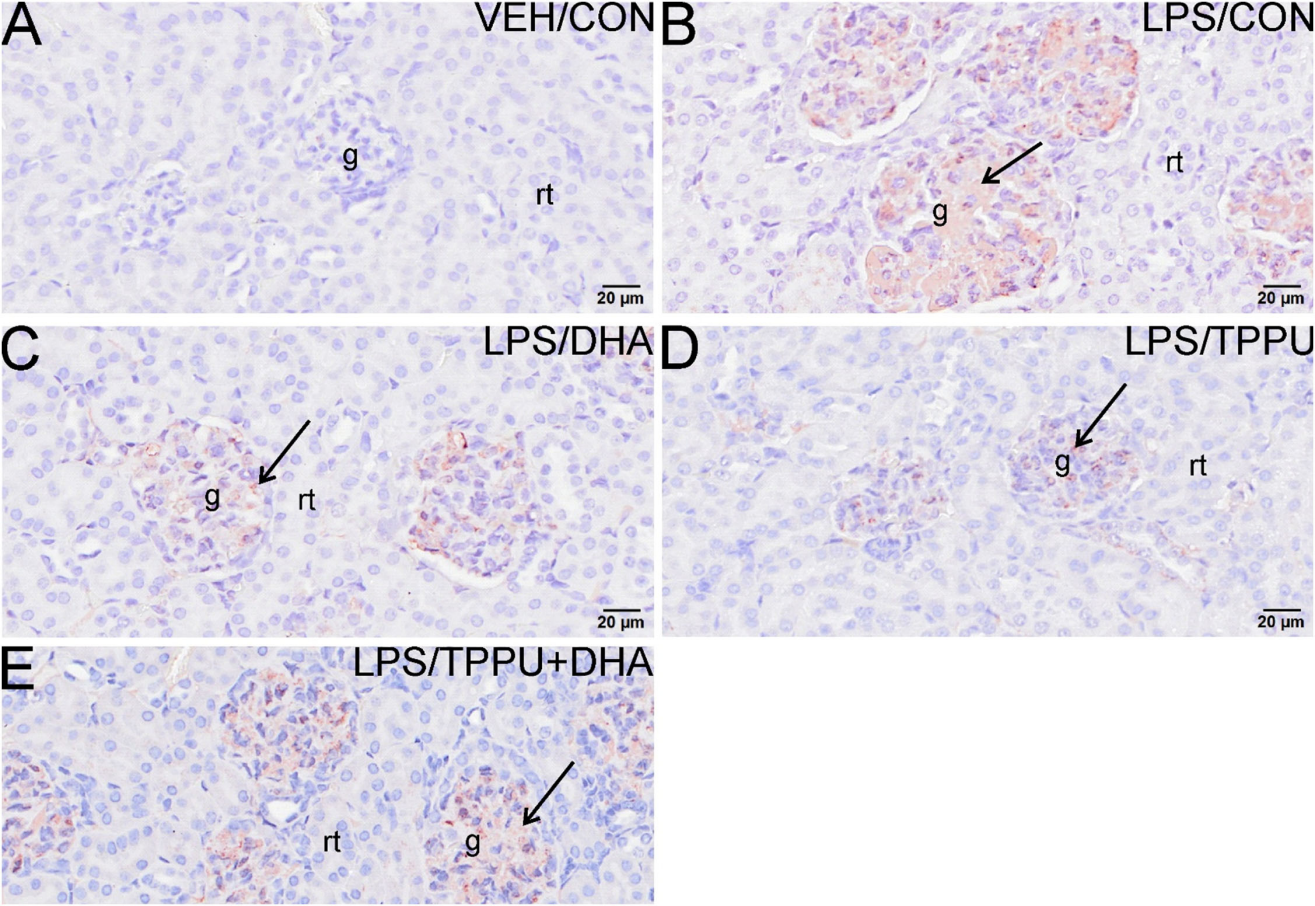
DHA alone and TPPU alone suppress R-LPS-induced IgG deposition in the kidney but are antagonistic when delivered together. Light photomicrographs of glomeruli immunohistochemically stained for IgG (arrows; brown chromogen) in kidneys from **(A)** vehicle (VEH)-treated/control diet (CON) mouse, **(B)** rough LPS-treated/CON mouse, **(C)** LPS/DHA mouse, **(D)** LPS/TPPU mouse, and **(E)** LPS/TPPU+DHA. No IgG+ staining in glomeruli of VEH/CON mouse **(A)**. Conspicuous IgG+ staining in medullary tissue of markedly enlarged glomeruli in LPS-treated mice **(B)**, LPS/DHA mice **(C)**, and LPS/TPPU+DHA mice **(E)**. Less medullary IgG+ staining in LPS/TPPU mouse **(D)** compared to other LPS-treated mice **(B, C, E)**. g, glomerulus; rt, renal tubule.

Histologic evaluation and scoring of PASH-stained kidney sections showed no evidence of GN in VEH/CON-treated mice (**Figures 9A, F**). Markedly hypertrophic and hypercellular glomeruli with thickened medullary membranes consistent with GN were observed in kidneys of LPS/CON and LPS/TPPU+DHA mice (**Figures 9B, E, F**), while less glomerular histopathology was evident in LPS/DHA and LPS/TPPU mice (**Figures 9C, D, F**). Consistent with histopathological findings, immunohistochemical evaluation of kidney sections stained with IgG-specific antibody similarly revealed that DHA alone and TPPU alone suppressed R-LPS-induced IgG deposition in the kidney but are antagonistic when delivered together (**Figures 10A–E**).

### DHA and TPPU modestly affect R-LPS-induced lymphoid cell accumulation in spleen and liver

**Figure 11:**
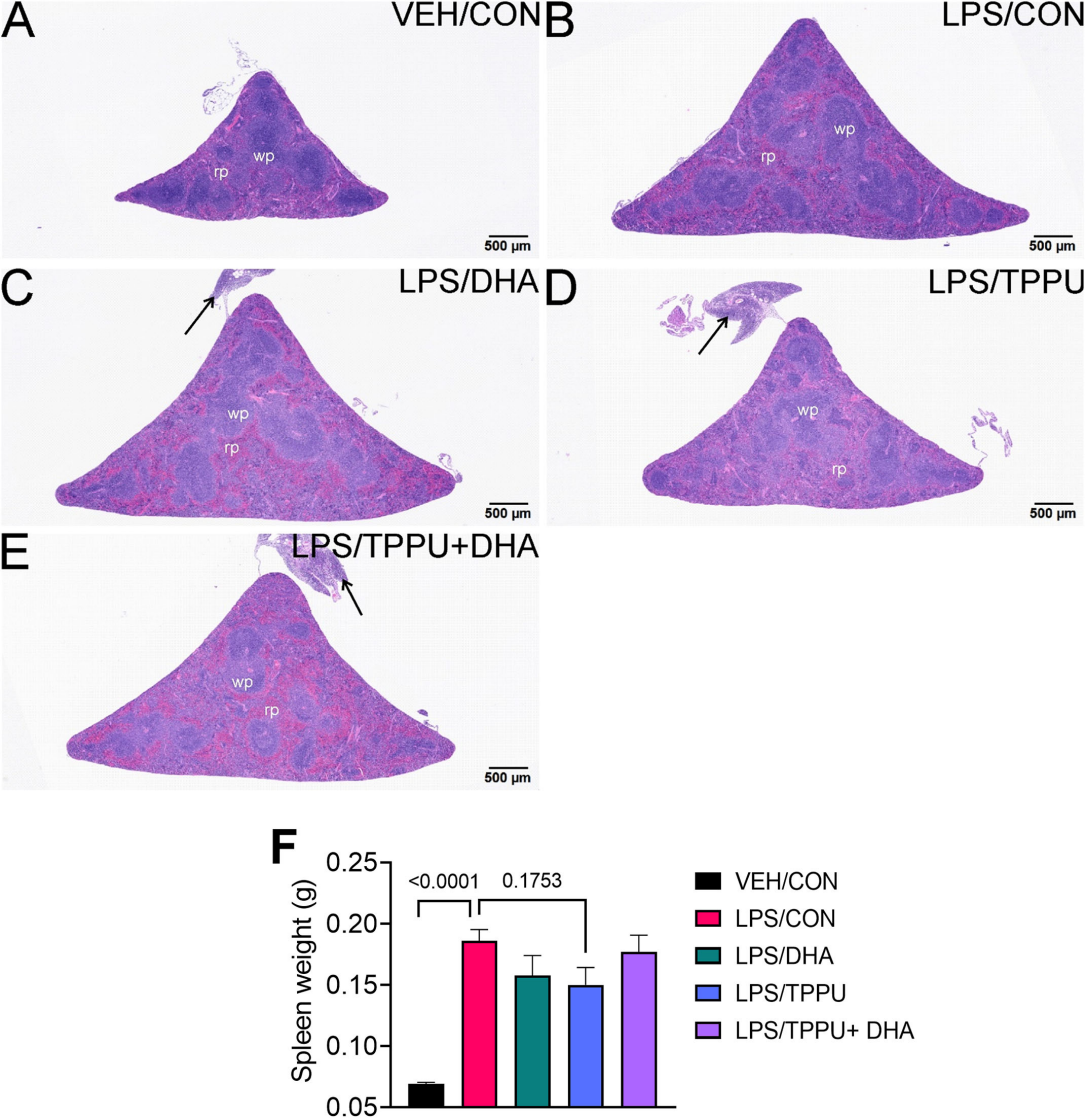
TPPU attenuates R-LPS-induced lymphoid hyperplasia in the spleen. Light photomicrographs of hematoxylin and eosin-stained tissue from the spleens of **(A)** vehicle (VEH)-treated/control diet (CON) mouse, **(B)** rough LPS-treated/CON mouse, **(C)** LPS/DHA mouse, **(D)** LPS/TPPU mouse, and **(E)** LPS/TPPU+DHA mouse. Spleens from LPS-treated mice **(B-E)** are enlarged due to lymphoid hyperplasia. Spleen from LPS/TPPU mouse **(D)** less enlarged than other LPS-treated mice. Mononuclear cell infiltration of peri-splenic fat (arrows) in LPS/DHA mice **(C)**, LPS/TPPU mice **(D)**, and LPS/TPPU+DHA mice **(E)**. **(F)** After 5 wk of biweekly i.p. LPS injections, mice were sacrificed, and spleens were weighed before additional tissue processing. Data are presented as mean ± SEM. wp, white pulp; rp, red pulp.

**Figure 12:**
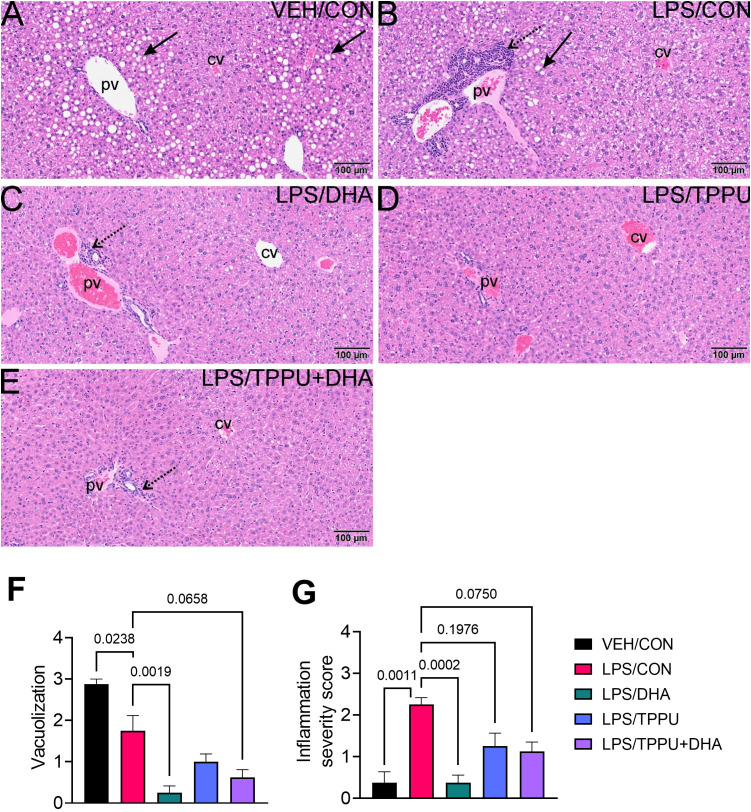
DHA suppresses R-LPS-induced liver inflammation and loss of vacuolization. Light photomicrographs of hematoxylin and eosin-stained tissue from the livers of **(A)** vehicle (VEH)-treated/control diet (CON) mouse, **(B)** rough LPS-treated/CON mouse, **(C)** LPS/DHA mouse, **(D)** LPS/TPPU mouse, and **(E)** LPS/TPPU+DHA mouse. Periportal hepatocellular vacuolization (solid arrow; characteristic of steatosis) in VEH/CON mice **(A)** and LPS/CON mice **(B)**. Lymphoid cell infiltration in periportal interstitial tissue (stippled arrow) in liver of LPS/CON mouse **(B)** with less hepatocellular vacuolization. Remainder of LPS-treated mice **(C-E)** have less periportal inflammatory cells and absence of hepatocellular vacuolization. Semi-quantitative scores for hepatic vacuolization **(F)** and inflammation severity **(G)**. Scoring was as follows: 0—no significant finding, 1—minimal, 2—mild, 3—moderate, 4—marked, 5—severe. Data are presented as mean ± SEM (*n =* 8). Values of p<0.1 are shown, with p<0.05 considered statistically significant. pv, periportal vein; cv, central vein; solid arrow, hepatocellular lipid vacuoles; stippled arrow, periportal cellular inflammation (predominantly mononuclear cells).

All H&E-stained splenic tissues from R-LPS- treated groups were enlarged due to lymphoid hyperplasia (**Figures 11A–E**). Splenic tissue from TPPU-fed mice had slightly less lymphoid hyperplasia then other LPS-treated mice (**Figure 11D**). Mean spleen weights were significantly elevated in the LPS/CON group compared to the VEH/CON group (**Figure 11F**). Mice fed DHA and TPPU diet exhibited trends toward reductions in spleen weight that were not statistically significant. Hematoxylin and eosin-stained liver sections were evaluated for histopathology (**Figure 12**). Periportal hepatocellular vacuolization was prominent in VEH/CON group possibly reflecting steatosis previously reported in NZBWF1 mice (**Figure 12A**). Less hepatocellular vacuolization with marked lymphoid cell infiltration was observed in periportal interstitial tissue in livers of LPS/CON mouse (**Figure 12B**). R-LPS-treated mice fed DHA, TPPU, and TPPU+DHA diet had less periportal inflammatory cells and absence of hepatocellular vacuolization (**Figures 12C–F**). Inflammatory severity scores were suppressed in DHA-fed mice with similar non-significant trends being observed in mice fed TPPU and TPPU+DHA diets (**Figure 12G**).

### DHA and/or TPPU do not affect R-LPS-induced autoantibody responses in plasma

Plasma from all mice within each experimental group were pooled and subjected to high-throughput autoantigen array for 120 IgG and IgM AAbs. While R-LPS robustly induced total and group-specific IgG and IgM autoantibodies in CON-fed mice, the magnitude of these responses was unaffected by DHA, TPPU, or TPPU+DHA treatments (**Supplementary Figure 4**).

### DHA and/or TPPU have limited impact on R-LPS-induced modulation of inflammatory and fatty acid metabolism gene expression in the kidney

We evaluated the impacts of DHA and TPPU on expression levels of inflammatory/autoimmune (i.e., *Il1b*, *Ccl2*, *Ccl7*, *Cxcl10*, *Cxcl13*, *C1qa*, *C3*, *Casp1*, *Tlr9*, *Tnfa*, *Tnfsf13b*) and fatty acid metabolism genes (i.e., *Alox15*, *Cyp2c44*, *Cyp2j6*, *Cyp2j9*, *Cyp2j11*, *Ephx1*, *Ephx2*, *Pla2g4a*, *Ptgs2*) genes in the kidney (**Supplementary Figure 5A**; **Supplementary Table 7**). R-LPS significantly induced expression of proinflammatory cytokines (i.e., *Il1b*, *Tnfa*), chemokines (i.e., *Ccl2*, *Ccl7*, *Cxcl13*), complement proteins (i.e., *C1qa*, *C3*), and other related (i.e., *Casp1*, *Tlr9*, and *Tnfsf13b*) genes relative to VEH/CON mice. Although *Cxcl10* mRNA was not significantly elevated by R-LPS, it demonstrated a modest increase compared to VEH/CON mice. Intriguingly, none of the selected inflammatory/autoimmune genes were significantly downregulated with DHA or TPPU, though some treatment groups exhibited therapeutic trends. For instance, LPS/DHA mice showed modest decreases in mRNA for *Ccl2*, *Ccl7*, *Cxcl13*, *C1qa*, *Casp1*, *Tlr9*, and *Tnfa* relative to LPS/CON mice. In addition, LPS/TPPU mice exhibited modest reductions in mRNA for *Ccl2*, *Ccl7*, *Casp1*, and *Tnfa*. Upon combining DHA and TPPU, the individual inhibitory effects of DHA and TPPU were diminished, with an exception to *Tlr9* expression which might be influenced only by DHA.

We found that R-LPS significantly reduced expression of several selected genes involved in lipid metabolite synthesis, including *Cyp2c44*, *Cyp2j6*, *Cyp2j9*, *Cyp2j11*, and *Ephx2*, compared to VEH-injected mice (**Supplemental Figure 5B**; **Supplementary Table 7**). In addition, R-LPS modestly downregulated *Ephx1*, *Pla2g4a*, and *Ptgs2*. In line with our observations for the inflammatory/autoimmune genes, we found that neither DHA nor TPPU significantly restored the expression levels of most fatty acid metabolism genes in our panel except for *Ephx2*, which was significantly upregulated in the LPS/DHA and LPS/TPPU+DHA groups compared to the LPS/CON group.

Other genes measured in our panel are noted in **Supplementary Table 7**. As anticipated, we observed significant upregulation of genes associated with kidney injury (i.e., *Ankrd1*, *Lcn2*) and oxidative stress (i.e., *Hmox*, *Nqo1*, *Sod2*) in LPS/CON mice relative to VEH/CON mice but found no significant expression level changes in DHA- and/or TPPU-treated mice. No significant changes in gene expression were noted for some type I IFN-regulated genes (i.e., *Irf7*, *Isg15*), but *Ifi44* expression was significantly reduced in all DHA-fed mice, regardless of TPPU consumption, relative to VEH/CON and LPS/CON mice.

## Discussion

LPS-accelerated autoimmune GN in NZBWF1 mice is increasingly being used as a preclinical model for identifying interventions applicable to preventing end-stage kidney disease associated with lupus (49, 51–54, 60, 76). We demonstrate here for the first time that the presence or absence of O-antigen polysaccharide profoundly influences the GN response and that R-LPS is required for optimal model performance. Compared to VEH and S-LPS, R-LPS caused significant weight loss associated with proteinuria, hematuria, histopathological scoring, glomerular IgG deposition, and influx of CD3^+^ and CD45R^+^ lymphocytes that might be associated with classical LPS-induced sickness behavior (77). When the effects of lipidome modulation by dietary DHA supplementation and/or sEH inhibition on R-LPS-accelerated GN were assessed, several novel findings were made. First, R-LPS treatment of CON-fed mice did not affect the red blood cell fatty acid profile but did reduce plasma concentrations of LA- and ARA-derived EpFAs/DiHFAs. Second, DHA supplementation skewed tissue PUFAs from ω-6 to ω-3 and shifted EpFAs/DiHFAs from primarily LA-/ARA-derived to EPA-/DHA-derived. Third, sEH inhibition with TPPU favored the accumulation of EpFAs over their respective vicinal diols. Fourth, based on proteinuria, hematuria, histopathologic scoring, and glomerular IgG deposition, the relative rank order of R-LPS-induced GN severity among groups fed experimental diets was: VEH/CON< R-LPS/DHA ≈ R-LPS/TPPU <<< R-LPS/TPPU+DHA ≈ R-LPS/CON. Fifth, DHA’s and TPPU’s effects on R-LPS-induced lymphocytic recruitment in spleen and liver were modest to negligible. Lastly, these interventions did not affect LPS-induced plasma AAb responses or kidney gene expression.

This investigation is the first to directly compare the efficacies of R-LPS and S-LPS in accelerating GN in lupus-prone mice. This effort was initiated after several failed preliminary attempts by our laboratory to induce GN with S-LPS. Several mechanisms have been proposed for LPS-accelerated GN including polyclonal B-cell activation, decreased efficiency of the mononuclear phagocyte system to uptake immune complexes, and/or delayed clearance of immune complexes from systemic circulation, all of which can contribute to increased deposits of immune complexes in the kidney (16–25). Consistent with polyclonal B cell activation, we observed that R-LPS but not S-LPS strongly induced germinal center expansion and splenomegaly, and, furthermore, R-LPS elicited a wide array of AAbs of the IgM and IgG isotypes. The mechanisms by which these two LPS chemotypes activate TLR4 are very different, and these differences may have special relevance to B cell activation. At low doses, S-LPS requires the glycosylphosphatidylinositol (GPI)-anchored co-receptor CD14 to trigger signal transduction through both MyD88-dependent and independent pathways, whereas, at low doses, R-LPS can initiate MyD88-dependent signaling in the absence of CD14 (57, 59). Thus, R-LPS efficiently activates TLR4 on both CD14^+^ and CD14^-^ cells as compared to S-LPS which acts primarily on CD14^+^ cells. Since B cells express TLR4 but lack CD14, it is tempting to speculate that they preferentially respond to R-LPS and not S-LPS, resulting in polyclonal activation that ultimately perpetuates AAb production and immune complex-driven GN in NZBWF1 mice. However, further studies are needed to test this and alternative hypotheses.

Red blood cells are commonly used as a surrogate to reflect tissue fatty acid profiles (31). As in our prior studies (63, 78), we found here that substitution of high oleic safflower oil in AIN-93G diets with DHA-containing microalgal oil increased DHA and EPA with nearly equivalent reductions of ARA. While some EPA might have resulted from DHA retroconversion, Metherel and coworkers (79) found that conversion of α-linolenic acid (ALA, C18:3ω3) to docosapentaenoic acid (DPA, C22:5ω3) by elongation/desaturation, mediated *via* feedback inhibition by DHA, resulted in the majority of EPA found in DHA-fed rats. Importantly, concurrent with elevated tissue concentrations of DHA and EPA, we observed decreases in plasma LA- and ARA-derived EpFAs and DiHFAs and corresponding increases in plasma EPA- and DHA-derived EpFAs and DiHFAs. Thus, consumption of marine ω-3 PUFAs alone can change blood levels of important bioactive CYP450 and sEH metabolites.

Preclinical (80–82) and clinical investigations (30, 32, 83, 84) generally support the premise that ω-3 PUFAs attenuate onset and progression of lupus-associated pathologic effects, including nephritis. Consistent with reported ameliorative actions of marine ω-3 PUFAs for preventing/treating chronic inflammatory and autoimmune diseases, we found here that consumption of DHA alone suppressed R-LPS-accelerated GN. Established mechanisms by which dietary intake of DHA and EPA potentially ameliorate systemic inflammation and downstream tissue damage include 1) modulating the structure and functionality of the plasma membrane and lipid rafts, 2) suppressed expression of proinflammatory cytokines, 3) binding with receptors, transcription factors, and enzymes at the expense of ω-6 PUFA binding, and 4) serving as substrates for highly pro-resolving ω-3 PUFA metabolites (reviewed by (30)). We have previously demonstrated in several macrophage models that ω-3 DHA displaces ω-6 ARA and ω-9 OA from the sn-2 position of membrane phospholipids, suppresses silica-induced expression of proinflammatory genes (e.g., *Nlrp3*, *Il1a*, *Il1b*) and type I IFN-regulated genes (e.g., *Irf7*, *Isg15*, *Oas2*, *Ifi44*), attenuates silica-triggered apoptotic and pyroptotic cell death, and enhances efferocytosis of cell corpses (85–87). Correspondingly, in recent studies using female NZBWF1 mice, we have found that DHA prevents silica-induced development of pulmonary ectopic lymphoid tissue (ELT) and downstream lupus GN, impedes expression of chemokine-related (e.g., *Cxcl9*, *Cxcl10*, *Ccr5*) and type I IFN-related (e.g., *Irf7*, *Isg15*, *Oas2*, *Ifi44*) genes in lung and kidney, and inhibits secretion of anti-nuclear AAb, proinflammatory cytokines (e.g., IL-1β, TNF-α, IL-6), chemokines, (e.g., BLC, MCP-5), enzymes (e.g., MMP-3, granzyme B), adhesion molecules (e.g., E-selectin, VCAM-1), co-stimulatory molecules (e.g., CD40L, CD48), and growth factors (e.g., IGF-1, Epiregulin) in BALF and plasma (78, 88–90). Furthermore, Cheng and coworkers have reported in both lupus-prone MRL/lpr mice and lupus patients that resolvin D1, a pro-resolving DHA metabolite, ameliorates disease progression by increasing Treg differentiation and decreasing Th17 differentiation from naïve CD4^+^ T cells (91). Thus, the pro-resolving effects of ω-3 PUFAs are multi-pronged and wide-reaching, with therapeutic importance in lupus and other chronic inflammatory/autoimmune pathologies.

Pharmacological effects of TPPU have been previously reported in many preclinical disease models (45, 46, 72, 92–102). In those studies, TPPU was delivered in drinking water in a polyethylene glycol (PEG) suspension, by oral gavage, or *via* injection. This investigation is the first to report the delivery of the sEH inhibitor TPPU in experimental rodent diet. Using this approach, we did not face issues associated with the low solubility of TPPU in water (0.06 mg/ml) (37), which was previously reported by Schmelzer and coworkers as a study limitation when administering AUDA, an sEH inhibitor with the same pharmacophore as TPPU, to LPS-challenged C57 mice (103). We estimate the daily dose of TPPU through diet to be 3 mg/kg/day which was sufficient to achieve plasma concentrations of approximately 5 µM (equivalent to 2000-fold of the Ki of TPPU) 4 wk after initiation of feeding. Our findings indicate that this dose was efficacious at significantly increasing the epoxide/diol ratio for LA. ARA, DHA, and EPA indicating robust inhibition of sEH. Accordingly, TPPU potently inhibits murine sEH and human sEH, with respective IC_50_ values 2.8 nM and 1.1 nM (34, 104) and respective Ki values of 2.5 nM and 0.64 nM (37). In addition, Liu and coworkers reported that TPPU exhibits a pharmacokinetic half-life (t_1/2_) of 37 ± 2.5 h in the blood following administration (3 mg/kg) to mice by oral gavage (36).

In this study, we hypothesized that cotreatment of ω-3 PUFA and sEH inhibitor would stabilize highly potent ω-3 EpFAs and therefore be more efficacious than the treatment of either ω-3 PUFA or sEH inhibitor alone. However, our results suggested that ω-3 PUFAs and the sEH inhibitor antagonize each other’s effects. Similar findings have been reported by Harris and coworkers (105) in which co-treatment of sEH inhibitor with DHA diminished the therapeutic effects of TPPU alone in a murine model of liver fibrosis. Although several studies suggest that ω-3 EpFAs are more potent in specific biological effects including angiotensin-II-dependent hypertension, nociception and autophagy, numerous studies have suggested that EpFAs generated from different PUFAs could play very different roles and the effects from two different subclasses of epoxy fatty acids could oppose each other (106–121). For example, EpDPE derived from ω-3 DHA is anti-angiogenic (112), but EpETrE derived from ω-6 ARA is proangiogenic (97, 122). Therefore, our results suggest that EpETrE could be a key lipid mediator for the anti-inflammatory, pro-resolving effects resulting from the treatment of sEH inhibitor TPPU, as it has been suggested by previous studies (108, 109, 114, 123). Our oxylipin analysis suggested that co-treatment of TPPU with DHA significantly decreases the endogenous level of EpETrE. Thus, DHA could potentially antagonize the effects of TPPU treatment alone.

While the absence of DHA and/or TPPU effects on LPS-induced inflammation-associated gene expression may suggest that these interventions interfere with a downstream event associated with GN development, such as immune complex deposition and associated kidney injury, transcriptomic data were only collected at termination and therefore may not reflect earlier timepoints. In support of this contention, we have found that DHA suppresses silica-induced inflammatory/autoimmune gene expression in NZBWF1 mice at 1, 5, and 9 wk after silica instillation but not at 13 wk post-instillation (88). Nevertheless, DHA suppressed silica-induced ELT neogenesis and GN at 13 wk (78). Thus, it will be important in future mechanistic studies of DHA and/or TPPU effects on R-LPS-induced GN to analyze blood biomarkers and tissues at multiple early timepoints.

One limitation of this study is that we focused primarily on phenotypic effects of DHA and/or TPPU on R-LPS-induced GN rather than underlying mechanisms. As Cavallo and coworkers previously reported in non-autoimmune and lupus-prone mice, R-LPS-induced GN may be the result of a cascade beginning with polyclonal B cell activation, then progressing to increased systemic AAb secretion, impaired clearance of immune complexes from circulation, and elevated immune complex deposition in the kidney (16–25). Although we found that R-LPS elicits toxicity in the kidney, spleen, and liver after 5 wk of i.p. injections, future studies should focus on elucidating the temporal mechanistic pathway by which R-LPS induces GN, with particular emphasis on evaluating toxicokinetic distribution of R-LPS from the peritoneum to downstream tissues, measuring impacts of R-LPS on polyclonal B cell activation, and determining whether R-LPS-induced kidney/spleen/liver toxicity occur dependently or independently of each other. Another constraint of this study is that we measured TPPU plasma concentration only after 5 R-LPS injections and not at any other timepoint to assess steady-state levels. TPPU has been shown to reach steady-state concentrations in the blood after 1-2 wk of oral administration by drinking water (124); however, it would be useful to collect plasma at multiple timepoints to confirm these findings in future studies involving dietary TPPU administration. A related limitation is that we focused only on sEH as a pharmacologic target, which is one of many epoxide hydrolases involved with PUFA metabolism (88). However, microsomal epoxide hydrolase is capable of significant EpETrE hydrolysis in sEH-knockout mice, suggesting that pharmacologic sEH inhibition does not completely block EpFA metabolism and therefore could affect experimental endpoints (124).

## Conclusion

Taken together, the results described herein show for the first time that absence of the O-antigenic polysaccharide in R-LPS is critical to accelerated GN in lupus-prone mice. While S-LPS elicited minimal toxicity in exposed mice, R-LPS triggered significant renal pathology characterized by proteinuria, hematuria, elevated BUN and plasma creatinine, glomerular damage, IgG deposition, and influx of CD3^+^/CD45R^+^ lymphocytes. Furthermore, lipidome modulation through DHA supplementation or sEH inhibition suppressed R-LPS-induced GN, but these ameliorative effects were greatly diminished upon combining the treatments. Separately, DHA and sEH inhibition delayed development of proteinuria and hematuria, dampened glomerular damage, and reduced glomerular IgG deposition with no significant effects on plasma autoantibody responses and expression of inflammatory and fatty acid metabolism genes in the kidney. Since it is currently unknown whether the perceived antagonistic relationship between DHA and TPPU is relevant only to our R-LPS mouse model or to other preclinical models for lupus as well, it will be essential in future investigations to evaluate how cotreatment with DHA and TPPU influence disease endpoints in other spontaneously-driven and environmentally-triggered lupus models. Additionally, it will be useful to investigate how direct administration of ω-3/6 EpFAs modulates pathologic biomarkers of R-LPS-induced autoimmunity in female NZBWF1 mice, versus coadministration with ω-3/6 PUFAs and sEH inhibitor. While our approach allowed us to broadly assess the effects of endogenous and DHA-derived EpFAs in R-LPS-induced GN, future investigations involving direct EpFA administration would provide valuable insight on specific EpFAs that may potentiate or prevent disease progression.

## Data availability statement

The datasets presented in this study can be found in online repositories. The names of the repository/repositories and accession number(s) can be found below: https://datadryad.org/stash, 10.5061/dryad.h44j0zppx.

## Ethics statement

The animal study was reviewed and approved by Institutional Animal Care and Use Committee (IACUC) at Michigan State University.

## Author contributions

OF: study design, coordination, feeding study, necropsy, data curation, data analysis/interpretation, figure preparation, manuscript preparation and submission. PC: study design, coordination, feeding study, necropsy, data curation, data analysis/interpretation, figure preparation, manuscript writing. EP: LC-MS/MS sample preparation, data analysis, manuscript writing. AE: LC-MS/MS sample preparation. JW: necropsy, lab analysis. RL: necropsy, lab analysis. JH: kidney/spleen/liver histopathology, data analysis, manuscript preparation. LH: AAb data acquisition/analysis, figure preparation. KL: study design, oversight, manuscript preparation. JP: study design, oversight, funding acquisition, data analysis/interpretation, manuscript preparation and submission. All authors contributed to the article and approved the submitted version.
