# Supplementary material for: Lipidome modulation by dietary omega-3 polyunsaturated fatty acid supplementation or selective soluble epoxide hydrolase inhibition suppresses rough LPS-accelerated glomerulonephritis in lupus-prone mice
Source: Front Immunol. 2023 Feb 16;14:1124910. doi: 10.3389/fimmu.2023.1124910 (PMC9978350; doi:10.3389/fimmu.2023.1124910)
Supplement: Supplementary file 1 [file DataSheet_1.pdf]

frontiers

Supplementary Material

Lipidome modulation by dietary omega-3 polyunsaturated fatty acid supplementation or selection soluble epoxide hydrolase inhibition suppresses rough LPS-accelerated glomerulonephritis in lupus-prone mice

Olivia K Favor[1,2†], Preeti S Chauhan[2,3†], Elham Pourmand[4], Angel M Edwards[4], James G Wagner[2,5], Ryan P Lewandowski[5], Lauren K Heine[1,2], Jack R Harkema[1,2,5], Kin Sing Stephen Lee[1,2,4], James J Pestka[2,3,6]

[1]Department of Pharmacology and Toxicology, College of Osteopathic Medicine, Michigan State University, East Lansing, Michigan, United States of America

[2]Institute for Integrative Toxicology, Michigan State University, East Lansing, Michigan, United States of America

[3]Department of Food Science and Human Nutrition, Michigan State University, East Lansing, Michigan, United States of America

[4]Department of Chemistry, Michigan State University, East Lansing, Michigan, United States of America

[5]Department of Pathobiology and Diagnostic Investigation, Michigan State University, United States of America

[6]Department of Microbiology and Molecular Genetics, Michigan State University, East Lansing, Michigan, United States of America

†These authors contributed equally to this work.

*** Correspondence:**

Olivia K. Favor, Department of Pharmacology and Toxicology, Michigan State University, East Lansing, MI 48824, U.S. Email: favoroli@msu.edu

James J. Pestka, Department of Food Science and Human Nutrition, Michigan State University, East Lansing, MI 48824, U.S. Email: pestka@msu.edu

1 Supplementary Tables

Supplementary Table 1. Study 1 experimental groups.

Experimental Group	Number of Animals (n)	LPS (-/+)	Experimental Diet
VEH/CON	2	-	AIN-93G
R-LPS/CON	4	+	AIN-93G
S-LPS/CON	4	+	AIN-93G

VEH, vehicle; CON, control; R-LPS, rough lipopolysaccharide; S-LPS, smooth lipopolysaccharide.

Supplementary Table 2. Study 2 experimental groups.

Experimental Group	Number of Animals (n)	LPS (-/+)	Experimental Diet
VEH/CON	8	-	CON (AIN-93G)
LPS/CON	8	+	CON (AIN-93G)
LPS/DHA	8	+	DHA (10 g/kg CON diet)
LPS/TPPU	8	+	TPPU (22.5 mg/kg CON diet)
LPS/TPPU+DHA	8	+	TPPU (22.5 mg/kg CON diet) + DHA (10 g/kg CON diet)

VEH, vehicle; CON, control; LPS, lipopolysaccharide; DHA, docosahexaenoic acid; TPPU, 1-(4-trifluoro-methoxy-phenyl)-3-(1-propionylpiperidin-4-yl) urea.

Supplementary Table 3. Study 2 experimental diet formulations.

	(g/kg total diet)			
	**CON**	**DHA**	**TPPU**	**TPPU+DHA**
**Carbohydrates**				
Corn starch	398	398	398	398
Maltodextrin (Dyetrose)	132	132	132	132
Sucrose	100	100	100	100
Cellulose	50	50	50	50
kcal (% of total)	63.2	63.2	63.2	63.2
**Proteins**				
Casein	200	200	200	200
L-Cysteine	3	3	3	3
kcal (% of total)	19.7	19.7	19.7	19.7
**Fats**[a]				
Corn oil[b]	10	10	10	10
High oleic-safflower oil[c]	60	35	60	35
DHA-enriched algal oil[d]	0	25[e]	0	25[e]
kcal (% of total)	17.1	17.1	17.1	17.1
**Other**				
AIN-93G mineral mix	35	35	35	35
AIN-93G vitamin mix	10	10	10	10
Choline bitartrate	3	3	3	3
TBHQ antioxidant	0.01	0.01	0.01	0.01
TPPU	0	0	0.0225	0.0225

All values are reported as mass (g) per kg of diet.
[a] As reported by the manufacturer
[b] Corn oil contained 612 g/kg linoleic acid and 26 g/kg oleic acid
[c] High oleic-safflower oil contained 750 g/kg oleic acid and 140 g/kg linoleic acid
[d] Algal oil contained 395 g/kg DHA and 215 g/kg oleic acid
[e] 10 g DHA/kg diet; calorically equivalent to human DHA consumption of 5 g/d

Supplementary Table 4. Study 2 experimental diet TPPU mass per kilogram of diet

	(mg TPPU / kg diet, mean ± SEM)	
	**TPPU**	**TPPU+DHA**
Expected Mass (mg/kg diet)	22.50	22.50
Measured Mass (mg/kg diet)	27.68 ± 3.85	32.80 ± 3.21

Data are presented as mg TPPU per kg experimental diet (mean ± SEM, n = 3) as measured by LC-MS/MS. TPPU was not measured in CON or DHA diets, as the expected mass was 0 mg/kg diet.

Supplementary Table 5. Waters TQ-XS tandem quadrupole UPLC/MS/MS linear gradient chromatographic method for analyte separation.

Mobile phase	A: 0.1% acetic acid in water	B: 84:16 acetonitrile/methanol + 0.1% acetic acid
**Gradient (minutes)**	**Percentage**	**Percentage**
Initial	65.0	35.0
1.00	60.0	40.0
3.00	45.0	55.0
8.50	35.0	65.0
12.50	28.0	72.0
15.00	18.0	82.0
16.00	0.0	100.0
18.10	65.0	35.0

Injection Volume: 10 µl
Flow Rate: 0.25 ml/min

Supplementary Table 6. Plasma oxylipin levels at necropsy as determined by LC-MS/MS.

		(concentration in nM, mean ± SEM)						
**PUFA Precursor**	**Metabolite**	**VEH/CON**	**LPS/CON**	**LPS/DHA**	**LPS/TPPU**	**LPS/TPPU+DHA**	**LOQ**	**LOD**
**LA**	9,10-EpOME	12.96±4.06	6.99±1.03^{A}	4.50±0.88^{A}	5.75±1.47^{A}	3.92±0.67^{A}	0.312	0.0468
**LA**	12,13-EpOME	17.62±5.81	8.85±1.31^{AB}	5.37±1.23^{A}	12.29±2.06^{B}	8.55±0.64^{AB}	0.312	0.0468
**LA**	9,10-DiHOME	11.82±6.15	5.87±0.61^{A}	2.91±0.61^{B}	4.94±0.66^{A}	2.79±0.30^{B}	0.630	0.0945
**LA**	12,13-DiHOME	4.97±1.83	3.47±0.48^{A}	2.04±0.63^{AB}	0.82±0.09^{B}	0.82±0.09^{B}	0.630	0.0945
**LA**	9-HODE	31.16±9.16	15.09±1.49^{A}	9.63±1.44^{B}	12.38±1.95^{A}	9.15±0.66^{B}	0.250	0.0375
**LA**	13-HODE	104.90±33.75	44.05±3.75^{A}	28.38±4.26^{B}	34.16±5.16^{A}	24.74±1.70^{B}	0.500	0.075
**LA**	9-oxo-ODE	8.48±4.07	2.52±0.53^{A}	0.50±0.35^{B}	1.21±0.53^{AB}	1.23±0.22^{AB}	0.500	0.075
**LA**	13-oxo-ODE	7.18±2.27	3.02±0.33*^{A}	1.74±0.32^{AB}	1.02±0.19^{B}	1.67±0.27^{AB}	0.312	0.0468
**LA**	EKODE	7.81±2.54	3.41±0.37^{A}	2.01±0.24^{AB}	1.47±0.28^{B}	1.81±0.16^{AB}	0.500	0.075
**DGLA**	15(S)-HETrE	1.24±0.59	0.13±0.13^{A}	<LOD^{A}	0.48±0.14^{B}	<LOD^{A}	0.125	0.0187
**ARA**	LTB4	1.74±0.92	0.16±0.3^{A}	0.11±0.07^{A}	0.06±0.06^{A}	0.52±0.04^{B}	0.250	0.0375
**ARA**	5,6-EpETrE	2.35±0.67	1.50±0.16^{A}	0.44±0.07^{B}	1.96±0.28^{A}	0.39±0.02^{B}	1.25	0.1875
**ARA**	8,9-EpETrE	7.35±2.02	2.99±0.96^{A}	2.06±1.06^{A}	3.09±0.43^{A}	6.91±4.14^{A}	1.25	0.1875
**ARA**	11,12-EpETrE	1.04±0.54	0.79±0.08^{A}	0.17±0.01^{B}	1.39±0.16^{A}	0.02±0.02^{B}	0.625	0.0937
**ARA**	14,15-EpETrE	2.17±0.65	1.44±0.22^{A}	0.08±0.08^{B}	2.59±0.27^{A}	0.11±0.07^{B}	0.625	0.0937
**ARA**	5,6-DiHETrE	1.06±0.11	1.24±0.12^{A}	0.20±0.05^{B}	0.48±0.02^{C}	0.15±0.03^{B}	0.250	0.0375
**ARA**	8,9-DiHETrE	16.26±1.91	8.18±0.50*^{A}	5.57±0.36^{B}	5.23±0.47^{B}	2.99±0.26^{C}	0.250	0.0375
**ARA**	11,12-DiHETrE	0.20±0.06	0.36±0.06*^{A}	<LOD^{B}	0.22±0.04^{AB}	<LOD^{B}	0.250	0.0375
**ARA**	14,15-DiHETrE	0.76±0.23	0.60±0.07^{A}	0.06±0.03^{BC}	0.21±0.01^{AB}	<LOD^{C}	0.630	0.0945
**ARA**	5-HETE	3.68±1.19	2.05±0.21^{A}	0.05±0.05^{B}	2.03±0.18^{A}	0.09±0.06^{B}	0.500	0.075
**ARA**	11-HETE	4.34±1.56	1.37±0.49^{A}	0.19±0.10^{B}	2.01±0.70^{A}	0.15±0.03^{B}	0.125	0.0187
**ARA**	12-HETE	306.30±102.3	77.58±49.40^{A}	25.94±16.94^{AB}	175.60±82.19^{AB}	9.05±4.12^{B}	0.500	0.075
**ARA**	15-HETE	7.08±3.06	1.67±0.73^{A}	0.12±0.12^{B}	3.11±1.16^{A}	<LOD^{B}	0.312	0.0468
**ARA**	19-HETE	<LOD	1.66±1.66^{A}	0.81±0.81^{A}	<LOD^{A}	<LOD^{A}	0.625	0.0937
**ARA**	5-oxo-ETE	1.46±0.99	0.10±0.10^{A}	0.05±0.05^{A}	0.29±0.15^{A}	<LOD^{A}	1.25	0.1875

**ARA**	12-oxo-ETE	5.28±2.97	0.41±0.41^{A}	<LOD^{A}	0.43±0.28^{A}	<LOD^{A}	1.25	0.1875
**ARA**	15-oxo-ETE	1.58±0.63	0.46±0.08^{A}	<LOD^{B}	0.18±0.06^{AB}	<LOD^{B}	0.250	0.0375
**ALA**	9,10-EpODE	0.24±0.11	0.21±0.08^{A}	<LOD^{B}	0.15±0.07^{AB}	0.02±0.02^{B}	0.312	0.0468
**ALA**	15,16-EpODE	1.13±0.31	0.81±0.10^{A}	0.27±0.12^{A}	1.01±0.27^{A}	0.75±0.10^{A}	0.250	0.0375
**ALA**	9,10-DiHODE	0.01±0.01	0.07±0.02^{AB}	<LOD^{A}	0.13±0.02^{B}	0.06±0.01^{AB}	0.630	0.0945
**ALA**	12,13-DiHODE	0.31±0.06	0.17±0.07^{A}	0.18±0.08^{A}	0.17±0.06^{A}	0.17±0.07^{A}	1.25	0.1875
**ALA**	15,16-DiHODE	<LOD	0.36±0.04^{A}	0.25±0.07^{AB}	0.01±0.01^{C}	0.05±0.02^{BC}	0.630	0.0945
**ALA**	9-HOTrE	1.23±0.22	0.69±0.07^{A}	0.42±0.07^{B}	0.37±0.08^{B}	0.43±0.03^{AB}	0.312	0.0468
**ALA**	13-HOTrE	1.30±0.45	0.26±0.13^{A}	0.11±0.01^{A}	0.12±0.08^{A}	<LOD^{A}	0.625	0.0937
**EPA**	11,12-EpETE	<LOD	<LOD^{A}	0.94±0.25^{B}	0.01±0.01^{A}	1.03±0.26^{B}	0.250	0.0375
**EPA**	14,15-EpETE	<LOD	<LOD^{A}	0.36±0.17^{AB}	<LOD^{A}	0.79±0.07^{B}	0.500	0.075
**EPA**	17,18-EpETE	<LOD	<LOD^{A}	2.20±0.33^{BC}	0.45±0.19^{AB}	5.33±0.41^{C}	1.25	0.1875
**EPA**	5,6-DiHETE	20.61±8.55	8.90±0.76^{A}	28.37±2.94^{B}	5.12±0.36^{C}	23.59±2.00^{B}	2.50	0.375
**EPA**	8,9-DiHETE	0.21±0.13	0.19±0.08^{A}	0.55±0.09^{B}	0.02±0.01^{A}	0.72±0.14^{B}	0.630	0.0945
**EPA**	11,12-DiHETE	<LOD	<LOD^{A}	0.37±0.07^{B}	<LOD^{A}	0.33±0.03^{B}	0.630	0.0945
**EPA**	14,15-DiHETE	<LOD	<LOD^{A}	0.46±0.07^{B}	0.02±0.01^{A}	0.25±0.03^{A}	0.630	0.0945
**EPA**	17,18-DiHETE	2.10±0.68	1.83±0.20^{AB}	16.25±2.52^{C}	1.17±0.07^{B}	6.98±0.58^{AC}	1.25	0.1875
**EPA**	5-HEPE	<LOD	<LOD^{A}	1.39±0.17^{B}	<LOD^{A}	1.60±0.10^{B}	0.625	0.0937
**EPA**	8-HEPE	0.10±0.10	<LOD^{A}	0.89±0.18^{B}	<LOD^{A}	0.54±0.13^{AB}	0.625	0.0937
**EPA**	12-HEPE	2.67±1.23	0.65±0.35^{A}	26.59±12.75^{B}	0.96±0.52^{A}	12.75±3.80^{B}	0.312	0.0468
**EPA**	15-HEPE	<LOD	0.02±0.02^{A}	0.73±0.37^{A}	<LOD^{A}	0.28±0.11^{A}	0.312	0.0468
**EPA**	18-HEPE	0.03±0.03	0.004±0.003^{A}	0.73±0.22^{B}	<LOD^{A}	0.74±0.04^{A}	0.625	0.0937
**EPA**	20-HEPE	<LOD	<LOD^{A}	1.56±0.13^{B}	<LOD^{A}	1.32±0.26^{B}	0.625	0.0937
**DHA**	7,8-EpDPE	0.43±0.30	0.10±0.07^{A}	3.59±1.17^{B}	0.33±0.17^{A}	2.18±0.45^{B}	1.25	0.1875
**DHA**	10,11-EpDPE	0.05±0.05	0.13±0.07^{A}	1.17±0.23^{B}	0.16±0.08^{A}	0.89±0.10^{B}	0.250	0.0375
**DHA**	13,14-EpDPE	0.25±0.17	0.11±0.07^{A}	1.93±0.48^{B}	0.29±0.12^{AC}	1.12±0.23^{BC}	0.625	0.0937
**DHA**	16,17-EpDPE	0.22±0.15	<LOD^{A}	2.49±0.85^{B}	0.21±0.09^{A}	2.04±0.35^{B}	1.25	0.1875
**DHA**	19,20-EpDPE	2.44±0.88	3.21±0.37^{A}	25.91±3.91^{A}	3.73±0.46^{A}	28.93±3.63^{A}	0.625	0.0937

**DHA**	7,8-DiHDPE	0.13±0.13	0.17±0.09^{A}	1.11±0.34^{A}	<LOD^{A}	<LOD^{A}	0.625	0.0937
**DHA**	10,11-DiHDPE	<LOD	<LOD^{A}	0.52±0.09^{B}	<LOD^{A}	0.19±0.04^{AB}	0.250	0.0375
**DHA**	13,14-DiHDPE	<LOD	<LOD^{A}	0.73±0.10^{B}	0.004±0.002^{A}	0.52±0.06^{B}	0.250	0.0375
**DHA**	16,17-DiHDPE	<LOD	<LOD^{A}	1.15±0.15^{B}	0.03±0.02^{AC}	0.46±0.03^{BC}	0.125	0.0187
**DHA**	19,20-DiHDPE	2.79±1.96	2.69±0.32^{A}	22.32±3.97^{B}	1.88±0.20^{A}	9.74±0.94^{B}	0.250	0.0375
**DHA**	20-HDHA	1.43±0.82	0.45±0.23^{A}	4.93±1.56^{B}	1.01±0.25^{A}	3.92±0.62^{B}	1.25	0.1875
**DHA**	22-HDHA	<LOD	<LOD^{A}	5.99±0.71^{B}	<LOD^{A}	4.28±0.65^{B}	0.625	0.0937

Data are presented as percent of total fatty acids (mean ± SEM, n = 8/gp) as measured by LC-MS/MS. Differences between VEH/CON and LPS/CON groups were compared by Student's t test. LPS/CON, LPS/DHA, LPS/TPPU, and LPS/TPPU+DHA groups were compared by ordinary one-way ANOVA followed by Tukey's *post-hoc* test. Nonparametric versions of these tests were used when applicable. Asterisks (*) indicate significant differences between VEH/CON and LPS/CON groups ($p<0.05$). Unique letters indicate significant differences between LPS/CON, LPS/DHA, LPS/TPPU, and LPS/TPPU+DHA groups ($p<0.05$). PUFA, polyunsaturated fatty acid; LOQ, limit of quantitation; LOD, limit of detection; LA, linoleic acid; DGLA, dihomo-gamma-linolenic acid; ARA, arachidonic acid; ALA, alpha-linolenic acid; EPA, eicosapentaenoic acid; DHA, docosahexaenoic acid.

Supplementary Table 7. Study 2 renal mRNA expression as determined by RT-PCR

	(Relative copy number, mean ± SEM)				
**Gene**	**VEH/CON**	**LPS/CON**	**LPS/DHA**	**LPS/TPPU**	**LPS/TPPU+DHA**
**Interleukins**					
Il1a	1.00±0.07	1.52±0.33^{A}	2.91±0.76^{A}	1.43±0.27^{A}	2.53±0.55^{A}
Il1b	1.00±0.10	6.31±0.99*^{A}	5.24±1.73^{A}	6.71±0.87^{A}	7.44±1.04^{A}
Il6	1.00±0.48	1.32±0.25^{A}	2.08±0.37^{A}	1.82±0.35^{A}	2.00±0.20^{A}
Il18	1.00±0.07	0.95±0.09^{A}	0.99±0.08^{A}	0.92±0.07^{A}	1.03±0.06^{A}
**Chemokines**					
Ccl2	1.00±0.12	6.91±1.13*^{A}	5.44±1.32^{A}	4.09±0.78^{A}	6.31±0.77^{A}
Ccl7	1.00±0.17	9.92±1.12*^{A}	7.45±1.56^{A}	5.42±1.04^{A}	7.57±1.11^{A}
Ccl12	1.00±0.18	2.52±0.31*^{A}	1.81±0.39^{A}	1.52±0.33^{A}	2.20±0.24^{A}
Cxcl9	1.00±0.15	1.19±0.17^{A}	0.96±0.28^{A}	0.72±0.11^{A}	0.97±0.14^{A}
Cxcl10	1.00±0.13	1.36±0.16^{A}	1.54±0.34^{A}	1.17±0.15^{A}	1.67±0.09^{A}
Cxcl13	1.00±0.14	13.68±2.35*^{A}	10.77±1.17^{A}	11.19±2.60^{A}	11.06±1.57^{A}
**Inflammation and Autoimmunity**					
C1qa	1.00±0.08	4.56±0.25*^{A}	3.44±0.30^{A}	3.99±0.36^{A}	4.13±0.37^{A}
C3	1.00±0.06	2.40±0.14*^{A}	2.16±0.14^{A}	2.45±0.34^{A}	2.56±0.36^{A}
Casp1	1.00±0.08	2.49±0.15*^{A}	1.89±0.12^{A}	2.05±0.16^{A}	2.36±0.21^{A}
Casp4	1.00±0.14	2.53±0.18*^{A}	1.90±0.23^{A}	2.23±0.20^{A}	2.63±0.21^{A}
Icam1	1.00±0.04	1.66±0.08*^{A}	1.57±0.12^{A}	2.05±0.15^{A}	1.86±0.11^{A}
Ifng	1.00±0.10	1.29±0.25^{A}	1.05±0.16^{A}	1.21±0.21^{A}	1.39±0.24^{A}
Lbp	1.00±0.06	1.12±0.03^{AB}	1.06±0.07^{A}	1.40±0.10^{B}	1.24±0.11^{AB}
Nfkb1	1.00±0.04	0.89±0.04^{A}	0.91±0.03^{A}	1.00±0.06^{A}	0.91±0.03^{A}
Nlrp3	1.00±0.21	1.59±0.18*^{A}	1.12±0.20^{A}	1.20±0.12^{A}	1.27±0.24^{A}
Nos2	1.00±0.15	1.36±0.10*^{A}	0.78±0.17^{B}	1.08±0.14^{AB}	1.19±0.19^{AB}
Pparg	1.00±0.20	1.02±0.15^{A}	0.71±0.07^{AB}	0.68±0.08^{AB}	0.60±0.03^{B}
Tlr4	1.00±0.03	1.40±0.11*^{A}	1.27±0.08^{A}	1.37±0.11^{A}	1.38±0.10^{A}
Tlr9	1.00±0.12	3.16±0.17*^{A}	2.13±0.29^{A}	2.81±0.32^{A}	2.32±0.18^{A}
Tnfa	1.00±0.17	5.45±0.88*^{A}	4.29±1.45^{A}	4.29±0.54^{A}	4.79±0.96^{A}
Tnfsf13b	1.00±0.12	2.21±0.10*^{A}	2.13±0.19^{A}	2.26±0.15^{A}	2.28±0.25^{A}
**Type I interferon-regulated genes**					
Ifi44	1.00±0.08	0.81±0.07^{AB}	0.67±0.04^{B}	1.01±0.08^{A}	0.71±0.07^{B}
Irf7	1.00±0.09	0.80±0.06^{A}	0.76±0.09^{A}	0.92±0.08^{A}	0.85±0.10^{A}
Isg15	1.00±0.12	0.89±0.07^{A}	0.95±0.14^{A}	1.10±0.09^{A}	1.16±0.07^{A}
Nlrc5	1.00±0.21	1.99±0.20*^{A}	1.33±0.17^{A}	1.80±0.19^{A}	1.41±0.15^{A}

Oas2	1.00±0.12	0.89±0.11^{A}	1.12±0.27^{A}	1.38±0.23^{A}	1.24±0.19^{A}
**Fatty Acid Metabolism**					
Alox15	1.00±0.27	0.80±0.15^{A}	0.89±0.08^{A}	0.80±0.23^{A}	1.34±0.21^{A}
Cyp2c44	1.00±0.04	0.53±0.03*^{A}	0.67±0.07^{A}	0.69±0.07^{A}	0.74±0.09^{A}
Cyp2j6	1.00±0.05	0.59±0.03*^{A}	0.67±0.04^{A}	0.77±0.07^{A}	0.74±0.08^{A}
Cyp2j9	1.00±0.06	0.63±0.06*^{A}	0.68±0.07^{A}	0.73±0.05^{A}	0.78±0.06^{A}
Cyp2j11	1.00±0.04	0.63±0.03*^{A}	0.80±0.07^{A}	0.82±0.07^{A}	0.72±0.07^{A}
Ephx1	1.00±0.05	0.77±0.07*^{A}	0.90±0.05^{A}	0.94±0.04^{A}	0.95±0.07^{A}
Ephx2	1.00±0.03	0.59±0.03*^{A}	0.93±0.07^{B}	0.77±0.06^{AB}	0.97±0.09^{B}
Pla2g4a	1.00±0.07	0.79±0.07*^{A}	0.82±0.06^{A}	0.85±0.07^{A}	0.91±0.07^{A}
Ptgs2	1.00±0.20	0.77±0.23^{A}	0.75±0.22^{A}	0.78±0.14^{A}	0.67±0.15^{A}
**Kidney Injury**					
Ankrd1	1.00±0.08	2.96±0.26*^{A}	2.77±0.36^{A}	2.48±0.25^{A}	3.43±0.49^{A}
Havcr1	1.00±0.07	1.00±0.13^{A}	1.11±0.23^{A}	2.00±0.35^{A}	1.11±0.21^{A}
Lcn2	1.00±0.13	2.78±0.48*^{A}	2.27±0.34^{A}	2.52±0.61^{A}	4.68±0.95^{A}
Tgfb1	1.00±0.06	1.66±0.10*^{A}	1.34±0.08^{A}	1.58±0.18^{A}	1.58±0.12^{A}
**Oxidative Stress**					
Hmox	1.00±0.06	1.79±0.07*^{A}	1.76±0.13^{A}	1.52±0.07^{A}	2.60±0.77^{A}
Ncf1	1.00±0.10	4.71±0.18*^{A}	3.47±0.29^{A}	3.67±0.30^{A}	4.23±0.56^{A}
Nqo1	1.00±0.05	0.55±0.03*^{A}	0.75±0.04^{A}	0.63±0.05^{A}	0.79±0.09^{A}
Sod2	1.00±0.04	0.58±0.02*^{A}	0.76±0.06^{A}	0.76±0.08^{A}	0.77±0.07^{A}

Gene expression data are presented as relative copy numbers (mean ± SEM, n = 8/gp) in relation to housekeeping genes. Differences between VEH/CON and LPS/CON groups were compared by Student's t test. LPS/CON, LPS/DHA, LPS/TPPU, and LPS/TPPU+DHA groups were compared by ordinary one-way ANOVA followed by Tukey's post-hoc test. Nonparametric versions of these tests were used when applicable. Asterisks (*) indicate significant differences between VEH/CON and LPS/CON groups ($p<0.05$). Unique letters indicate significant differences between LPS/CON, LPS/DHA, LPS/TPPU, and LPS/TPPU+DHA groups ($p<0.05$).

Supplementary Table 8. List of Key Reagents, Chemicals, and Kits

Reagent	Vendor	Catalog Number	Lot Number
AIN-93G Purified Rodent Diet without Vitamin Mix	Dyets Inc.	110700	
AIN-93G VX Vitamin Mix	Dyets Inc.	310025	
LouAna Safflower Oil	LouAna Oils		
Mazola Corn Oil	Mazola		
Microalgal Oil Containing 40% DHA	DHASCO		
TPPU	Synthesized in-house		
S-LPS from *Salmonella enterica* serotype minnesota	Sigma Aldrich	L6261	059M4137V
R-LPS from *Salmonella enterica* serotype minnesota Re 595	Sigma Aldrich	L9724	0000099960
Urine Reagent Strip (Glucose-Protein) Rapid Test Kit	Cortez Diagnostics	URS-2P	82721
Urine Reagent Strip-1B (URS-1B) Blood	Teco Diagnostics	URS-1B	87703
Urea Nitrogen (BUN) Colorimetric Detection Kit	Thermo Fisher Scientific	EIABUN	
Creatinine (serum) Colorimetric Assay Kit	Cayman Chemical	700460	0602046
Polyclonal Goat Anti-IgG Antibody	Bethyl Labs	A-90-100A	
Polyclonal Rabbit Anti-Mouse CD3 Antibody	Abcam	ab5690	
Monoclonal Rat Anti-Mouse CD45R Antibody	Becton Dickinson	550286	
RNeasy Mini Kit	Qiagen	74104	

2 Supplementary Figures

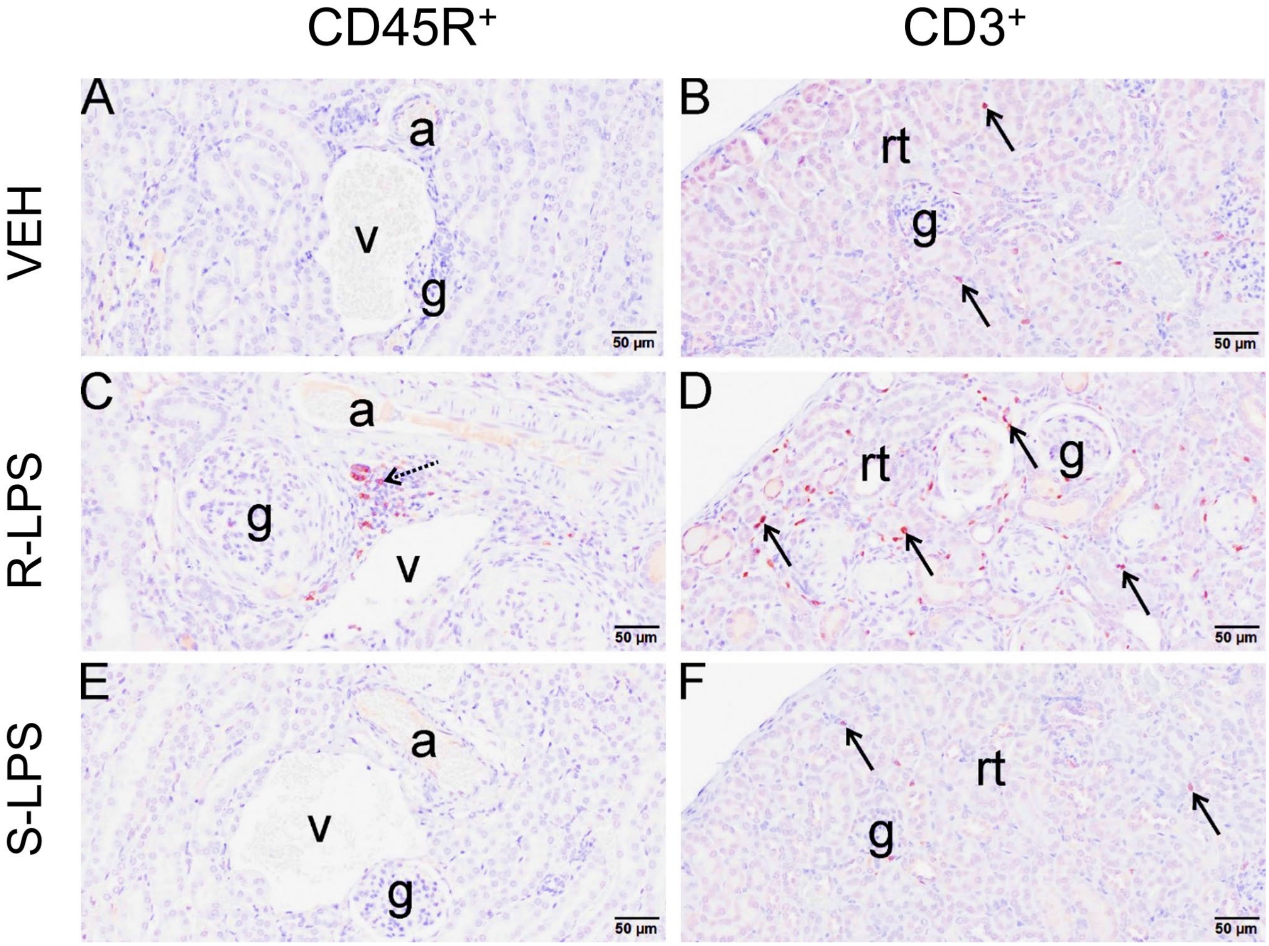

Supplementary Figure 1. R-LPS but not S-LPS induces B and T cell accumulation in kidney. Light photomicrographs of cortical tissues from kidneys of mice that received i.p. injections of saline vehicle (VEH) alone **(A, B)**, rough (R)-LPS **(C, D)** and smooth (S)-LPS **(E, F)**. Tissues were immunohistochemically stained for CD45R+ lymphoid B cells (stippled arrows) **(A, C, E)** or CD3 lymphoid T cells (solid arrows) **(B, D, F)**, and counter stained with hematoxylin. a, cortical artery; v, cortical vein; g, glomerulus; rt, renal tubule.

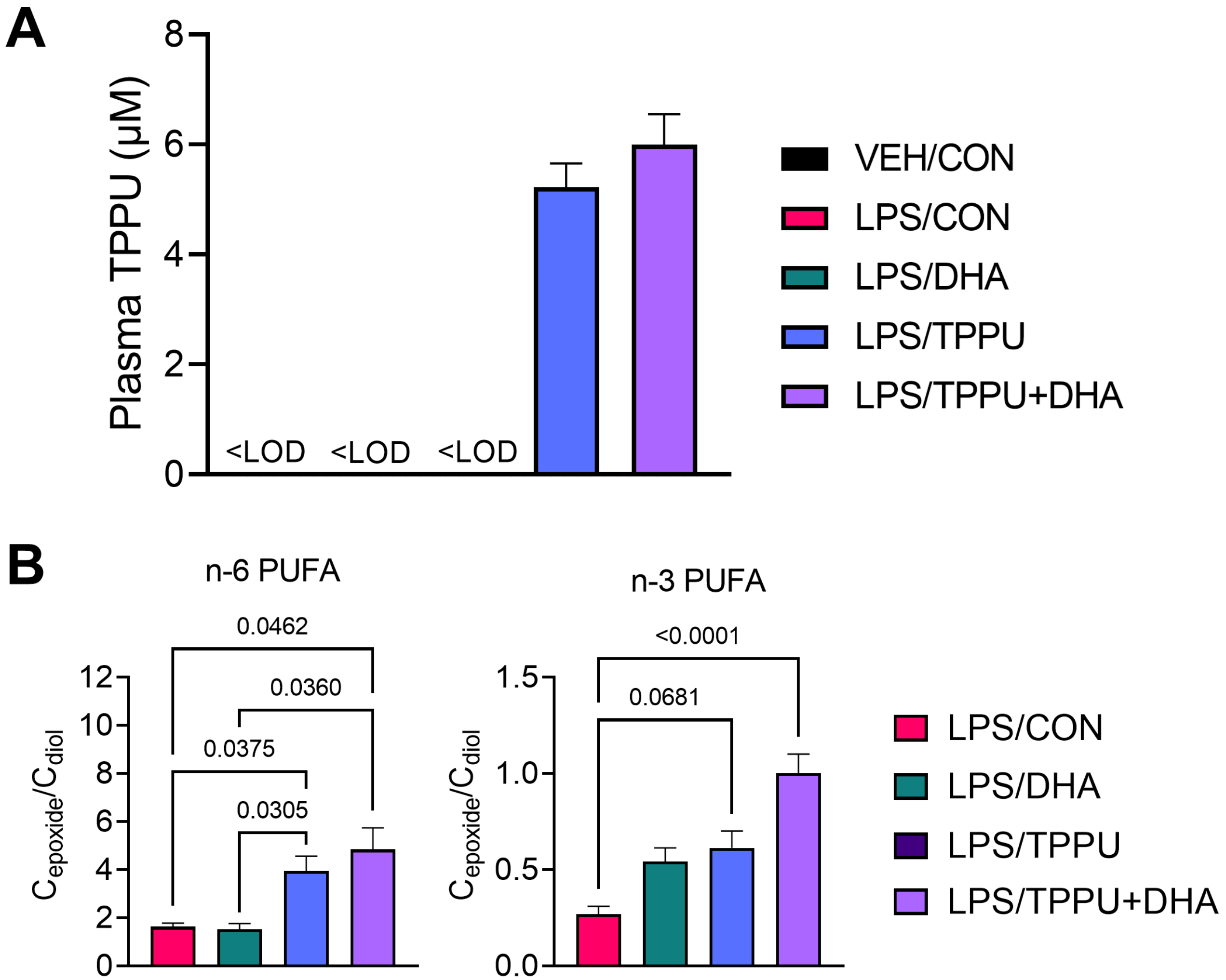

Supplementary Figure 2. TPPU delivery via diet increases the drug in plasma and inhibits sEH. (A) TPPU delivered by dietary supplementation is efficiently transferred to plasma. Plasma concentration of TPPU was measured at 10 wk of age by LC-MS/MS. Data are presented as mean ± SEM. <LOD = below limit of detection. (B) Supplementation with DHA and/or TPPU modulates plasma omega-6 and omega-3 epoxide/diol metabolite ratios in LPS-injected NZBWF1 mice. Administration of TPPU separately and with DHA significantly increases epoxide/diol ratios of pooled omega-6 metabolites (i.e., LA, ARA) and pooled omega-3 metabolites (i.e., EPA, DHA). Data are presented as mean ± SEM (n = 8). Values of $p<0.1$ are shown, with $p<0.05$ considered statistically significant.

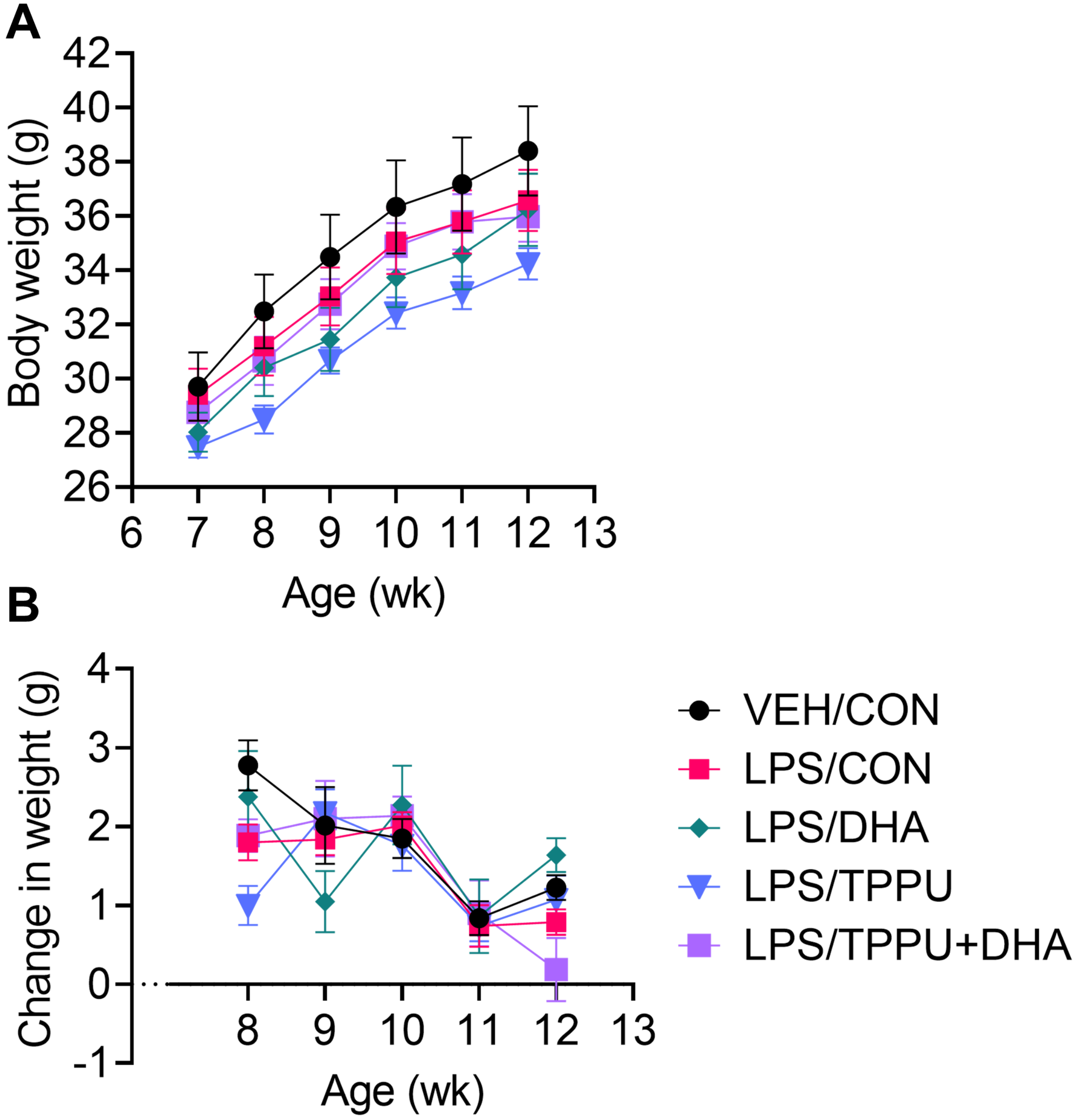

Supplementary Figure 3. Dietary DHA and/or TPPU supplementation does not significantly affect body weight and weight gain over time. (A) Mice were weighed weekly, concurrently with the first LPS injection of the wk. Data are presented as mean ± SEM. (B) weekly changes in body weight were calculated by taking the difference between body weight one wk and body weight the subsequent wk. Data are presented as mean ± SEM.

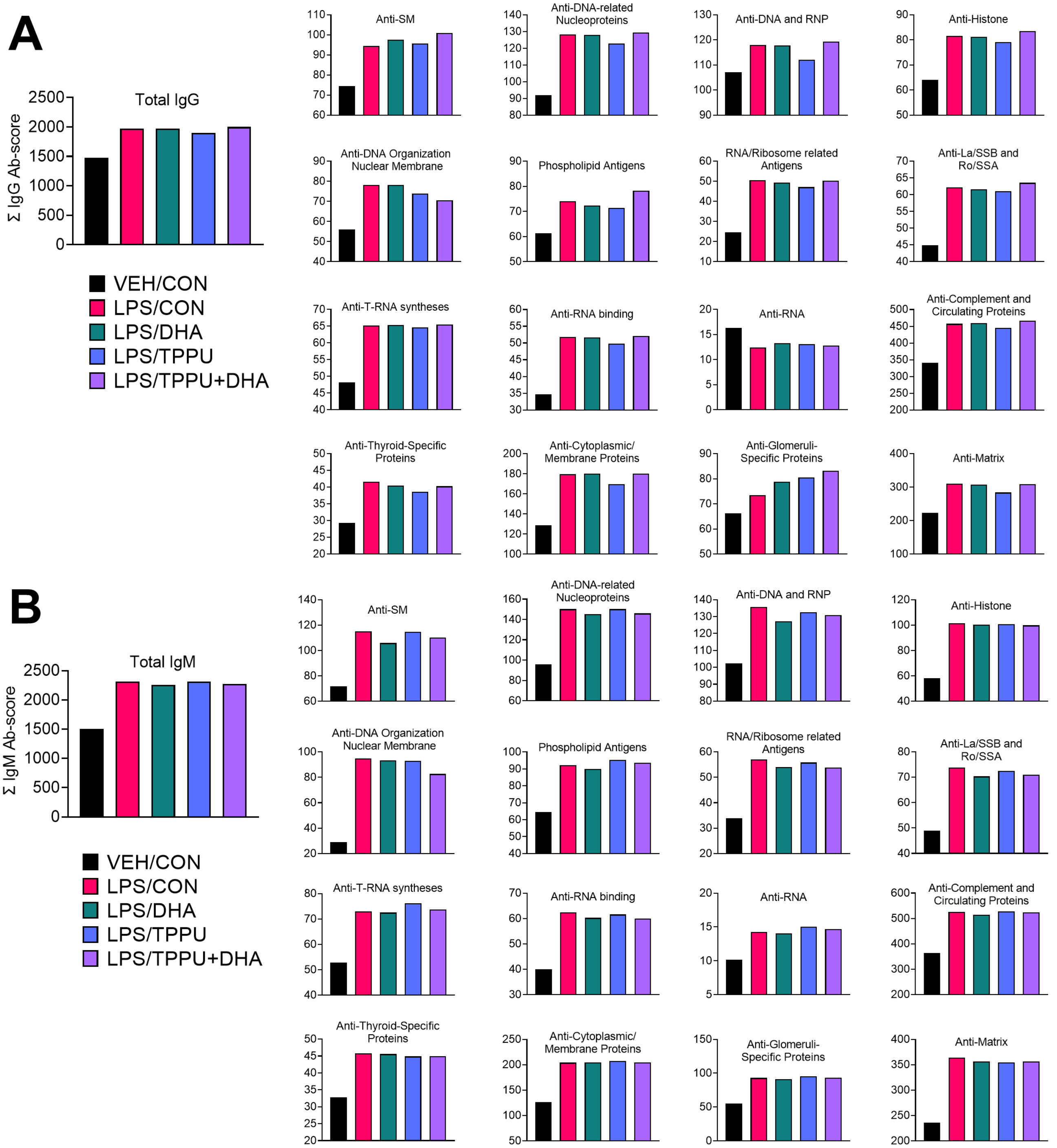

Supplementary Figure 4. Broad spectrum of IgG and IgM autoantibodies (AAbs) induced by R-LPS are largely unaffected by DHA and/or TPPU. Plasma from all individuals within each experimental group (n=8/gp) were pooled and 122 IgG and IgM AAbs measured by high-throughput AAb array. Antibody scores (Ab-scores) were calculated for total and specified (A) IgG and (B) IgM AAbs. Data for total AAbs depicted as Σ IgG Ab-score and Σ IgM Ab-score, respectively. Data for specified AAbs depicted as individual IgM and IgM Ab-scores, respectively.

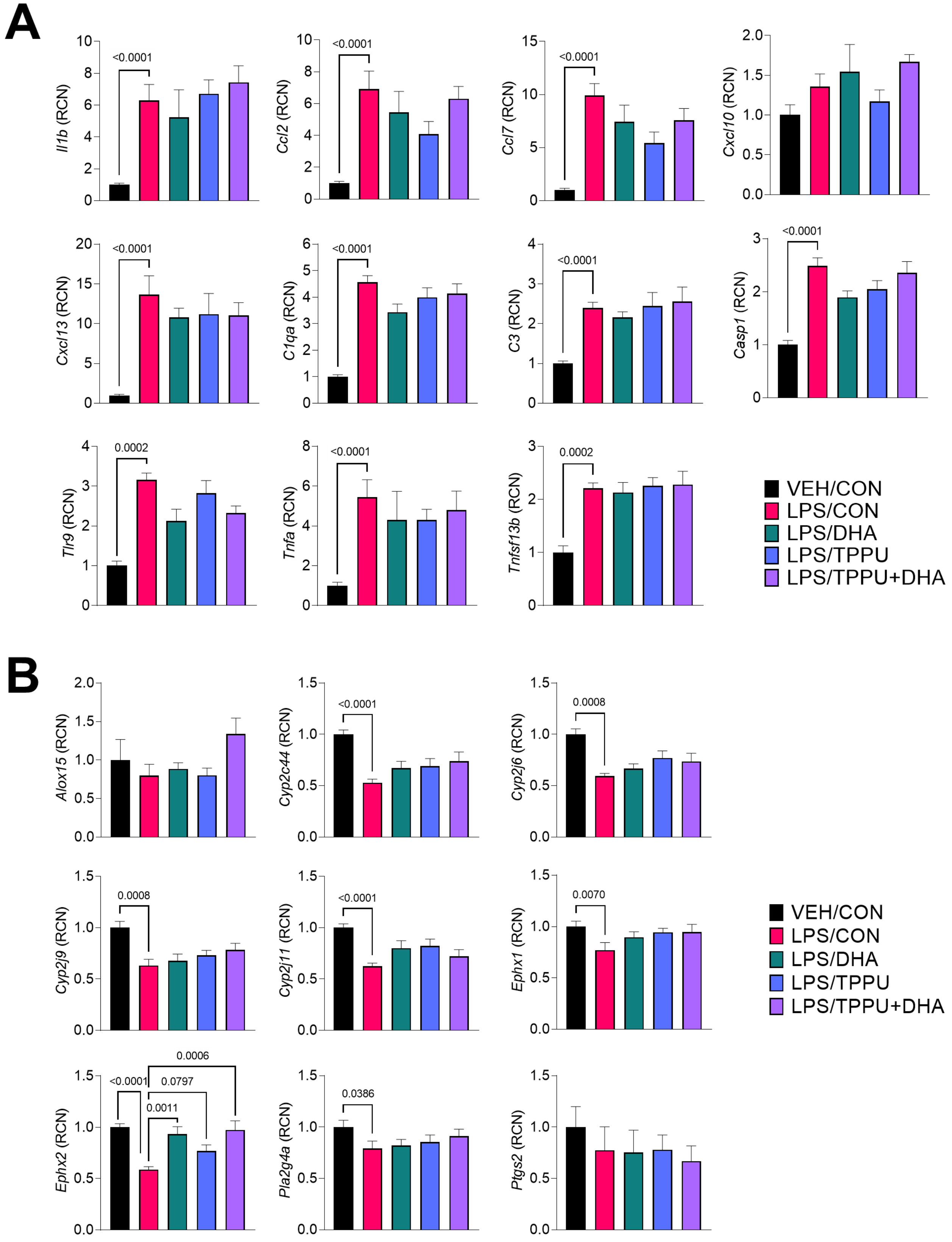
A
Il1b (RCN)
<0.0001
Ccl2 (RCN)
<0.0001
Ccl7 (RCN)
<0.0001
Cxcl10 (RCN)
Cxcl13 (RCN)
<0.0001
C1qa (RCN)
<0.0001
C3 (RCN)
<0.0001
Casp1 (RCN)
<0.0001
Tlr9 (RCN)
0.0002
Tnfa (RCN)
<0.0001
Tnfsf13b (RCN)
0.0002
VEH/CON
LPS/CON
LPS/DHA
LPS/TPPU
LPS/TPPU+DHA
B
Alox15 (RCN)
Cyp2c44 (RCN)
<0.0001
Cyp2j6 (RCN)
0.0008
Cyp2j9 (RCN)
0.0008
Cyp2j11 (RCN)
<0.0001
Ephx1 (RCN)
0.0070
Ephx2 (RCN)
<0.0001
0.0006
0.0797
0.0011
Pla2g4a (RCN)
0.0386
Ptgs2 (RCN)
VEH/CON
LPS/CON
LPS/DHA
LPS/TPPU
LPS/TPPU+DHA

Supplementary Figure 5. Representative inflammatory and fatty acid metabolism genes modulated by R-LPS in kidney are largely unaffected by DHA and/or TPPU. Following sacrifice, kidneys were isolated and analyzed for RNA expression of selected (A) inflammatory/autoimmune genes (i.e., *Il1b*, *Ccl2*, *Ccl7*, *Cxcl10*, *Cxcl13*, *C1qa*, *C3*, *Casp1*, *Tlr9*, *Tnfa*, *Tnfsf13b*) and (B) fatty acid metabolism genes (i.e., *Alox15*, *Cyp2c44*, *Cyp2j6*, *Cyp2j9*, *Cyp2j11*, *Ephx1*, *Ephx2*, *Pla2g4a*, *Ptgs2*). Data are presented as mean ± SEM (n = 6-8). Values of $p<0.1$ are shown, with $p<0.05$ considered statistically significant.
